# Gastric cancer biomarker analysis in patients treated with different adjuvant chemotherapy regimens within SAMIT, a phase III randomized controlled trial

**DOI:** 10.1038/s41598-022-12439-3

**Published:** 2022-05-20

**Authors:** Takashi Oshima, Akira Tsuburaya, Kazuhiro Yoshida, Takaki Yoshikawa, Yohei Miyagi, Yasushi Rino, Munetaka Masuda, Jia Guan, Patrick Tan, Heike I. Grabsch, Junichi Sakamoto, Shiro Tanaka

**Affiliations:** 1 Department of Gastrointestinal Surgery, Kanagawa Cancer Center, 2-3-2 Nakao, Asahi-ku, Yokohama, Kanagawa 241-8515 Japan; 2 Department of Surgery, Ozawa Hospital, 1-1-17, Honcho, Odawara, Kanagawa 250-0012 Japan; 3 Department of Surgical Oncology, Gifu University Graduate School of Medicine, 1-1 Yanagito, Gifu, Gifu 501-1194 Japan; 4 Department of Gastric Surgery, National Cancer Center Hospital, 5-1-1 Tsukiji, Chuo-ku, Tokyo, 104-0045 Japan; 5 Kanagawa Cancer Center Research Institute, 2-3-2 Nakao, Asahi-ku, Yokohama, Kanagawa 241-8515 Japan; 6 Department of Surgery, Yokohama City University, 3-9 Fukuura, Kanazawa-ku, Yokohama, Kanagawa 236-0004 Japan; 7 Department of Clinical Biostatistics, Graduate School of Medicine, Kyoto University, Yoshida Konoe-cho Sakyo-ku, Kyoto, Kyoto 606-8501 Japan; 8 Cancer Science Institute of Singapore, National University of Singapore, 14 Medical Drive, Singapore, 117599 Singapore; 9 Department of Pathology, GROW School for Oncology and Reproduction, Maastricht University Medical Center+, Maastricht, The Netherlands; 10 Division of Pathology and Data Analytics, Leeds Institute of Medical Research at St James’s, University of Leeds, Leeds, UK; 11 Tokai Central Hospital, 4-6-2 Sohara Higashijimacho, Kakamigahara, Gifu 504-8601 Japan

**Keywords:** Tumour biomarkers, Gastric cancer

## Abstract

Biomarkers for selecting gastric cancer (GC) patients likely to benefit from sequential paclitaxel treatment followed by fluorinated-pyrimidine-based adjuvant chemotherapy (sequential paclitaxel) were investigated using tissue samples of patients recruited into SAMIT, a phase III randomized controlled trial. Total RNA was extracted from 556 GC resection samples. The expression of 105 genes was quantified using real-time PCR. Genes predicting the benefit of sequential paclitaxel on overall survival, disease-free survival, and cumulative incidence of relapse were identified based on the ranking of p-values associated with the interaction between the biomarker and sequential paclitaxel or monotherapy groups. Low *VSNL1* and *CD44* expression predicted the benefit of sequential paclitaxel treatment for all three endpoints. Patients with combined low expression of both genes benefitted most from sequential paclitaxel therapy (hazard ratio = 0.48 [95% confidence interval, 0.30–0.78]; p < 0.01; interaction p-value < 0.01). This is the first study to identify *VSNL1* and *CD44* RNA expression levels as biomarkers for selecting GC patients that are likely to benefit from sequential paclitaxel treatment followed by fluorinated-pyrimidine-based adjuvant chemotherapy. Our findings may facilitate clinical trials on biomarker-oriented postoperative adjuvant chemotherapy for patients with locally advanced GC.

## Introduction

In Japan, 134,650 patients were diagnosed with gastric cancer (GC) in 2019, out of which 25,850 had stage II/III disease, according to the Union for TNM 8th edition^1,2^. The standard treatment for patients with stage II/III GC in Japan is curative D2 gastrectomy followed by postoperative adjuvant chemotherapy^3^, based on the results of the Japanese Adjuvant Chemotherapy Trial of S-1 for Gastric Cancer (ACTS-GC) and Korean Adjuvant capecitabine and oxaliplatin for gastric cancer after D2 gastrectomy (CLASSIC) randomized phase III trials^4–7^. However, despite the improved overall survival (OS) with adjuvant chemotherapy, the five-year OS rate of patients with pathological stage III (pStage III) GC remains unsatisfactory. Hence, there is an urgent clinical need to develop new more effective regimens and personalized adjuvant chemotherapy treatments based on biomarkers.

It has been reported recently that patients with curatively resected pathological (p) stage III GC treated with adjuvant docetaxel and S-1 had significantly longer 3-year recurrence-free survival than those treated with adjuvant S-1 monotherapy chemotherapy (JACCRO GC-07 study) ^8^. Based on the results, chemotherapy with S-1 and docetaxel after D2 gastrectomy was recommended as the new standard of care for patients with pStage III GC in Japan.

Biomarkers for personalized adjuvant chemotherapy have been investigated in resected cancer tissue specimens from the ACTS-GC and CLASSIC trials ^9–13^. Although several novel GC biomarkers were discovered in ACTS-GC, none of the biomarkers showed a significant interaction with S-1 treatment ^9–12^. In a post-hoc analysis of resection specimens from the CLASSIC trial, it was reported that the combined RNA expression levels of three genes (granzyme B [GZMB], WARS, and caudal-related homeobox [CDX1]) were able to predict the benefit of adjuvant chemotherapy with capecitabine plus oxaliplatin compared to no adjuvant chemotherapy^13^.

In addition to fluorinated-pyrimidine plus platinum-based anticancer drugs such as capecitabine plus oxaliplatin, fluorinated-pyrimidines plus taxanes such as paclitaxel or docetaxel have been considered for GC treatment ^14^. Taxane-based anticancer drugs have lower incidences of nephrotoxicity or neuropathy than platinum-based compounds, such as cisplatin or oxaliplatin, and can be administered safely in an outpatient setting. Both the JACCRO GC-07 trial and SAMIT have demonstrated improved outcomes in the subgroup of patients with pathological stage III GC treated with postoperative adjuvant chemotherapy using fluorinated-pyrimidine and taxane-based anticancer drugs ^8,14^. However, the disease recurrence rate within 2 years after surgery was 75.3% in the JACCRO GC-07 trial and 55.3% in the SAMIT trial. Therefore, adjuvant chemotherapy using fluorinated-pyrimidine plus taxane-based anticancer drugs may only be effective in a subset of GC patients. If such patients can be identified by biomarker assessment in the gastrectomy specimens, adjuvant chemotherapy regimens could be personalized, and patient outcomes could be improved.

In the present study, we performed a post-hoc analysis of tissue samples collected from patients recruited in the SAMIT using a comprehensive panel of mRNA expression-based biomarkers. The aim of the present study was to identify genes suitable for selecting patients likely to benefit more from adjuvant chemotherapy with sequential paclitaxel followed by fluorinated-pyrimidine (sequential paclitaxel).

## Results

### Patients and sample collection

**Figure 1:**
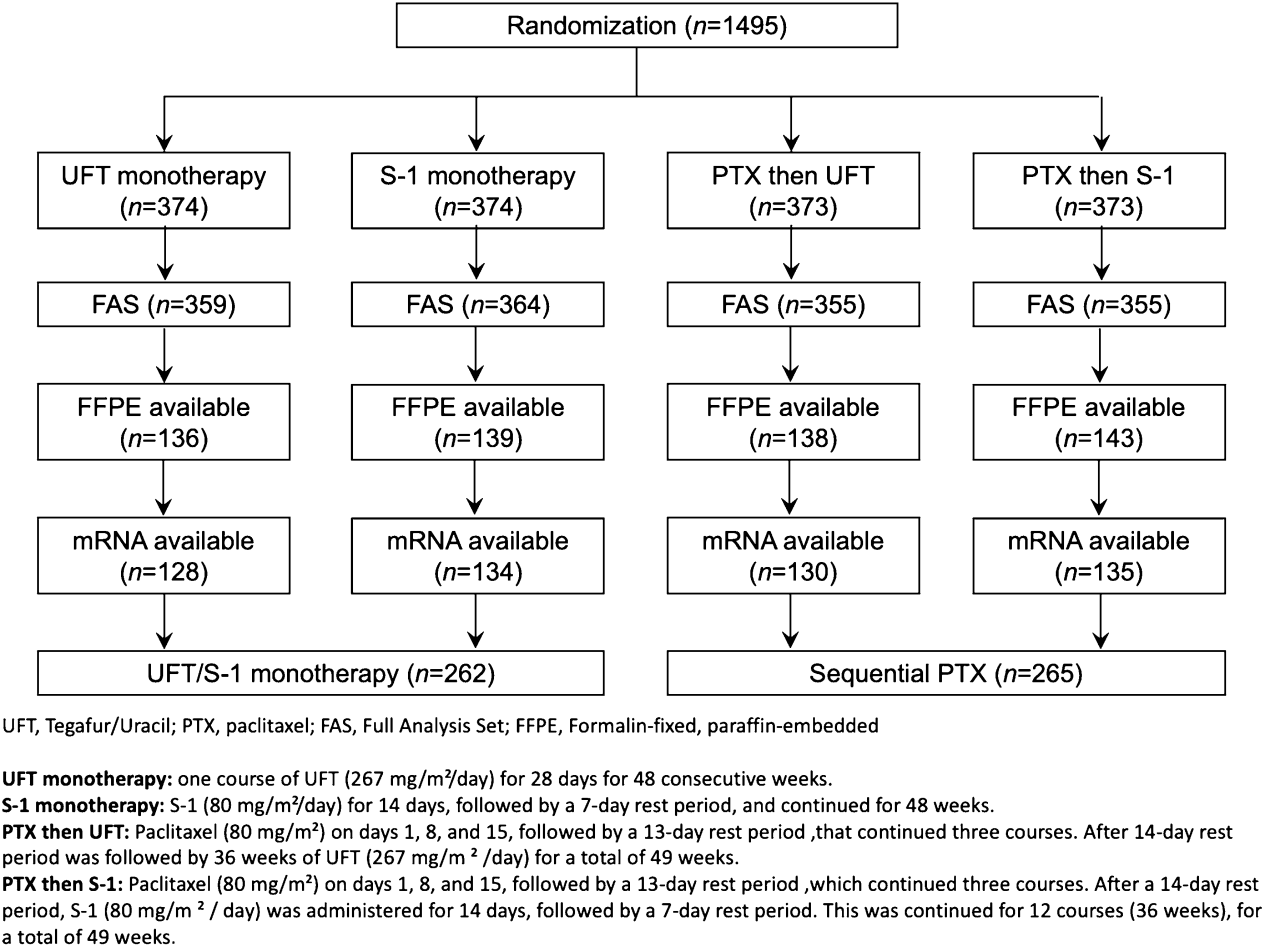
Flowchart of SAMIT patients available for primary analysis and subsequent biomarker analysis. Formalin-fixed, paraffin-embedded (FFPE) samples were available from 556 SAMIT patients. Twenty-nine patients had to be excluded owing to insufficient RNA.

Formalin-fixed paraffin-embedded (FFPE) samples were retrospectively collected from 556 SAMIT patients. Twenty-nine patients had to be excluded subsequently due to insufficient RNA, leaving 527 patients for biomarker analysis (Fig. 1). The clinicopathological characteristics of the patients included in the current study were representative of the entire SAMIT population (Table 1). Except for sex (there were more males in the sequential paclitaxel treatment group [p = 0.04]), the clinical and pathological characteristics were well balanced between the sequential paclitaxel treatment and fluorinated-pyrimidine monotherapy subgroups (Supplementary Table S1, Online Resource 1). The median follow-up times from randomization were 56.8 (interquartile range [IQR] = 45.3–69.8 months) and 59.1 months (IQR = 46.2–72.8 months) for patients in the fluorinated-pyrimidine monotherapy and sequential paclitaxel arms, respectively.

**Table 1:** Clinical and pathological characteristics of patients included in the biomarker analysis compared to the entire SAMIT patient cohort.

	Biomarker analysis cohort (*n* = 527)		Entire SAMIT cohort (*n* = 1433)		*p*-value
	No. of patients	%	No. of patients	%	
**Arms**					0.980
S-1 only	128	24.3	359	25.1	
UFT only	134	25.4	364	25.4	
Paclitaxel then UFT	130	24.7	355	24.8	
Paclitaxel then S-1	135	25.6	355	24.8	
**Age**					1.00
< 65 years	243	46.7	670	46.8	
≥ 65 years	284	53.3	763	53.2	
**Sex**					1.00
Male	361	68.5	980	68.4	
Female	166	31.5	453	31.6	
**PS**					0.211
0	442	83.9	1234	86.1	
1	85	16.1	199	13.9	
2 or 3	0	0	0	0	
**Tumor location**					
T	12	2.3	43	3.0	0.785
U	131	24.9	366	25.5	
M	176	33.4	482	33.6	
L	208	39.5	542	37.8	
**Tumor diameter**					0.554
< 65	278	52.8	686	47.9	
≥65	249	47.2	747	52.1	
**Surgery**					0.432
Total gastrectomy	241	45.7	696	48.6	
Proximal gastrectomy	1	0.2	5	0.3	
Distal gastrectomy	284	53.9	728	50.8	
**Lymph node dissection**					0.252
D1	1	0.2	2	0.1	
D1 +	22	4.2	92	6.4	
D2	496	94.1	1311	91.5	
D3	8	1.5	28	2.0	
**Lauren’s classification**					0.791
Intestinal type	212	40.2	567	39.6	
Diffuse type	315	59.8	866	60.4	
**pT**					0.051
1	7	1.3	12	0.8	
2	161	30.6	366	25.5	
3	339	64.3	966	67.4	
4	20	3.8	89	6.2	
**pN**					0.167
0	109	20.7	268	18.7	
1	90	17.1	296	20.6	
2	119	22.6	350	24.4	
3	209	40.0	519	36.2	
**pTNM stage**					0.080
I	37	7.0	77	5.4	
IIA	107	20.3	266	18.6	
IIB	106	20.1	318	22.2	
IIIA	105	19.9	344	24.0	
IIIB	101	19.2	291	20.3	
IIIC	71	13.5	147	10.3	

*UFT* tegafur/uracil, *T* total stomach, *U* upper third of stomach, *M* medium third of stomach, *D* distal third of stomach, *T* pathological tumor depth, *pN* pathological lymph node metastasis, *M* distant metastasis, *PS* performance status.

### Predictive biomarkers for selecting patients likely to benefit from sequential paclitaxel therapy

We conducted multivariable Cox regression analysis to assess the potential relationships between gene expression level and overall survival (OS), disease-free survival (DFS), or cumulative incidence of relapse after sequential paclitaxel therapy; the genes were ranked based on the interaction-related *p*-values. *Visinin-like 1* (*VSNL1*) and *CD44* were the only genes with mRNA expression levels that were statistically significant as predictive biomarkers of sequential paclitaxel treatment for all three endpoints (Supplementary Table S2, Online Resource 1).

**Figure 2:**
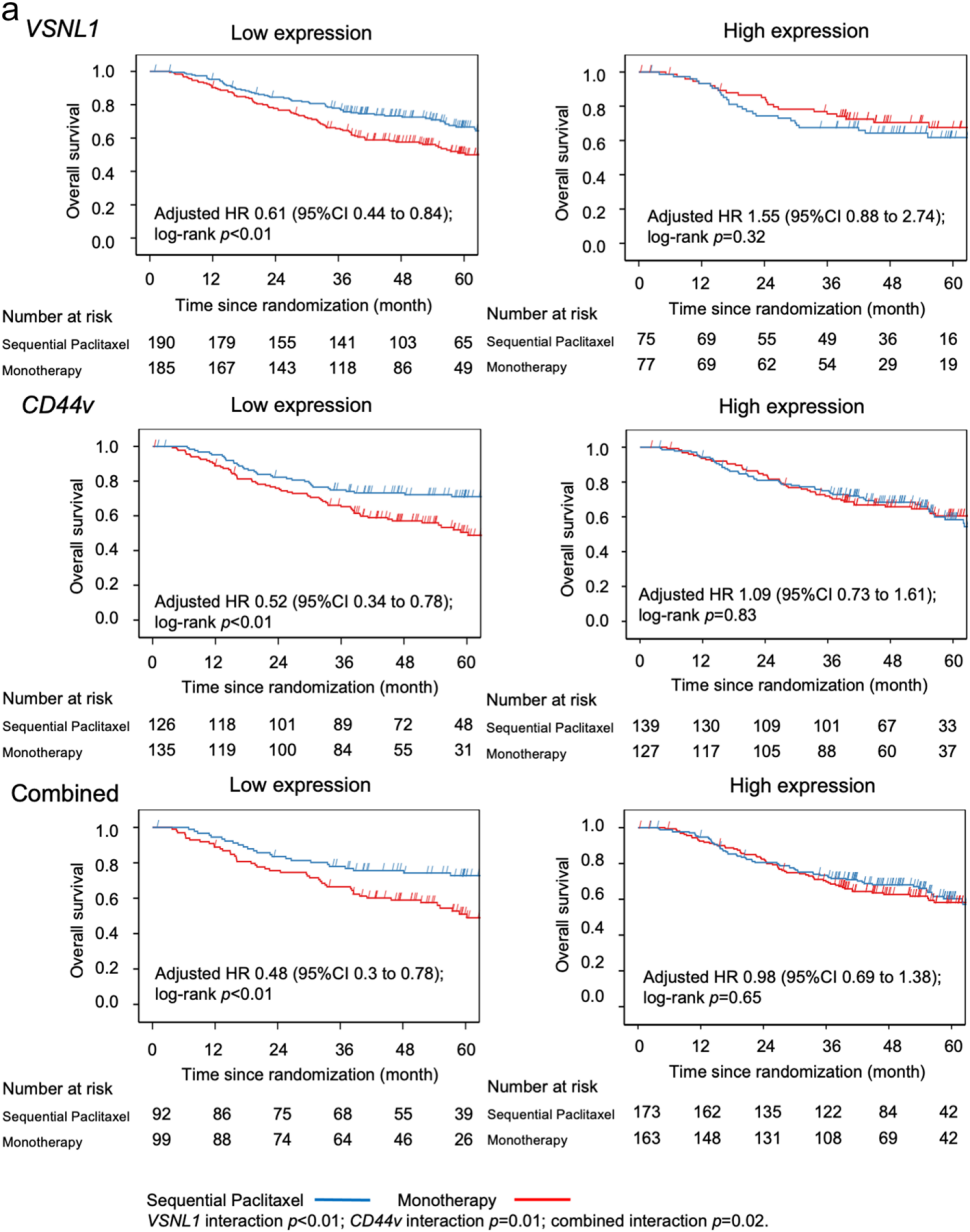

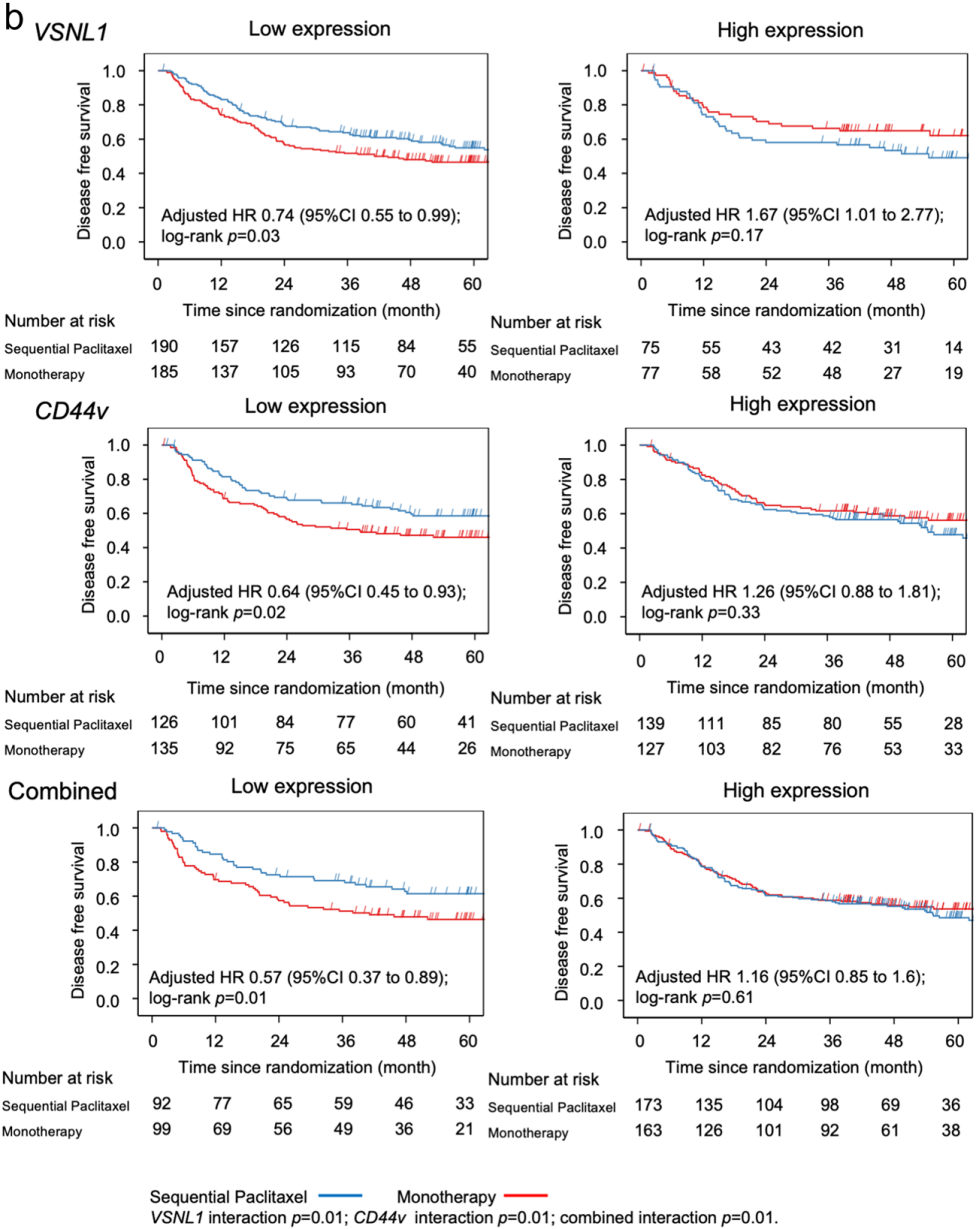

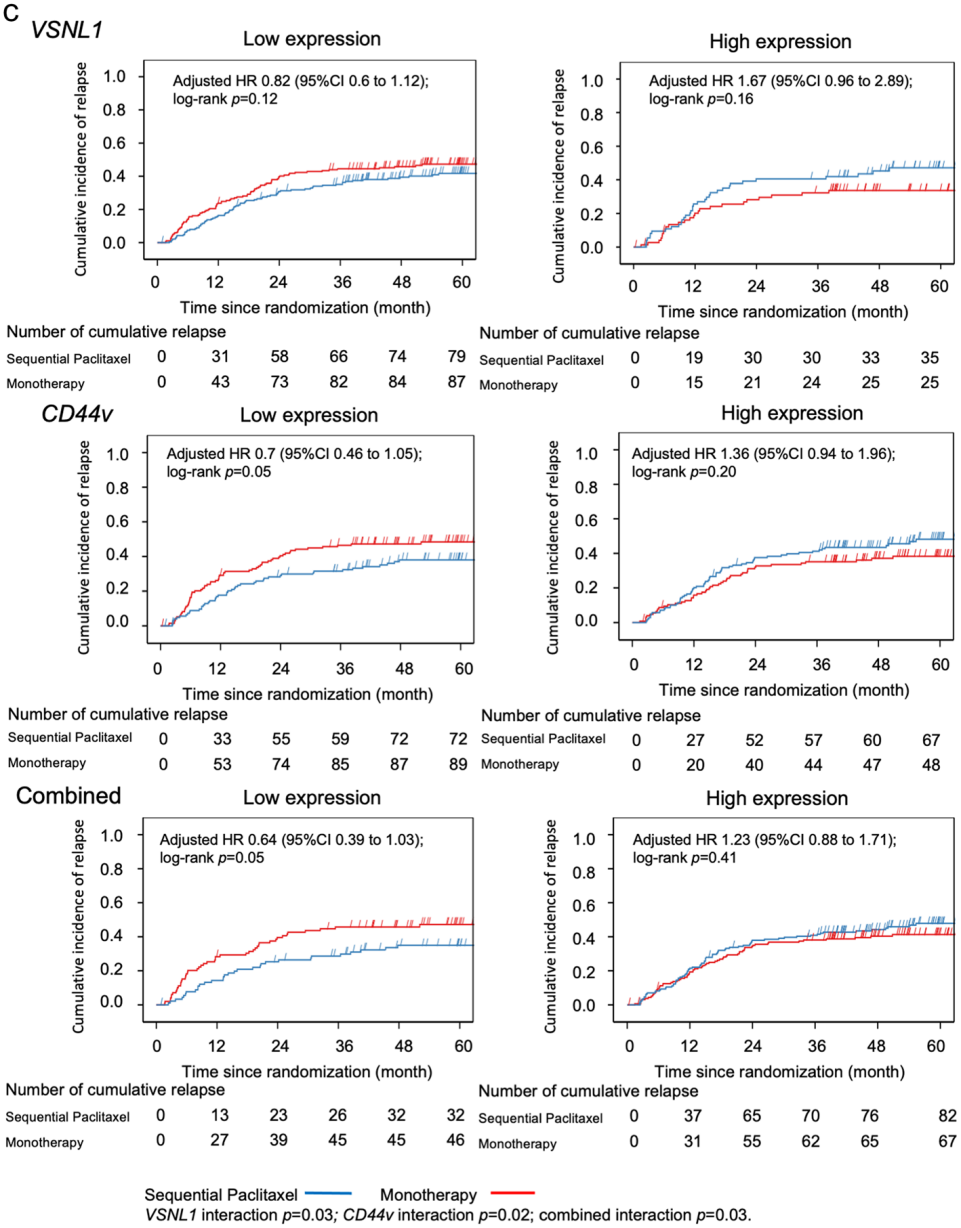
Kaplan–Meier curves based on gene expression level in the sequential paclitaxel and monotherapy arms. Patients with low RNA expression levels of *VSNL1*, *CD44*, or both had significantly longer overall survival (**a**), longer disease-free survival (**b**), and lower cumulative incidence of relapse (**c**) after sequential paclitaxel treatment than after monotherapy.

A total of 191 (36.2%) patients showed combined low expression of both genes, which was associated with the greatest benefit from sequential paclitaxel treatment compared to fluorinated-pyrimidine monotherapy (Table 2). Patients with low levels of expression of *VSNL1*, *CD44v*, or both, had significantly longer OS and DFS after sequential paclitaxel treatment than after monotherapy (Fig. 2a,b). However, no such effect was observed in the cumulative incidence of relapse (Fig. 2c).

**Table 2:** Effects of sequential paclitaxel followed by UFT or S-1 on overall survival, disease-free survival, and cumulative incidence of relapse, based on gene expression levels.

	Subgroups	Comparison of sequential paclitaxel and monotherapy over time				
		HR	95% CI		Main effect *p*	Interaction *p*-value
**Overall survival**						
Total	(*n* = 527)	0.76	0.57	1.01	0.05	
*VSNL1*	Low expression (*n* = 375)	0.61	0.44	0.84	< 0.01	< 0.01
	High expression (*n* = 152)	1.55	0.88	2.74	0.13	
*CD44*	Low expression (*n* = 261)	0.52	0.34	0.78	< 0.01	0.01
	High expression (*n* = 266)	1.09	0.73	1.61	0.67	
Combined	low expression of both genes (*n* = 191)	0.48	0.3	0.78	< 0.01	0.02
	high expression of either gene (*n* = 336)	0.98	0.69	1.38	0.89	
**Disease-free survival**						
Total	(*n* = 527)	0.91	0.7	1.17	0.44	
*VSNL1*	Low expression (*n* = 375)	0.74	0.55	0.99	0.04	0.01
	High expression (*n* = 152)	1.67	1.01	2.77	0.05	
*CD44*	Low expression (*n* = 261)	0.64	0.45	0.93	0.02	0.01
	High expression (*n* = 266)	1.26	0.88	1.81	0.21	
Combined	low expression of both genes (*n* = 191)	0.57	0.37	0.89	0.01	0.01
	high expression of either gene (*n* = 336)	1.16	0.85	1.6	0.35	
**Cumulative incidence of relapse**						
Total	(*n* = 527)	0.98	0.75	1.28	0.87	
*VSNL1*	Low expression (*n* = 375)	0.82	0.6	1.12	0.21	0.03
	High expression (*n* = 152)	1.67	0.96	2.89	0.07	
*CD44*	Low expression (*n* = 261)	0.7	0.46	1.05	0.08	0.02
	High expression (*n* = 266)	1.36	0.94	1.96	0.1	
Combined	low expression of both genes (*n* = 191)	0.64	0.39	1.03	0.07	0.03
	high expression of either gene (*n* = 336)	1.23	0.88	1.71	0.22	

*HR* hazard ratio, *CI* confidence interval, *UFT* tegafur/uracil.

**Figure 3:**
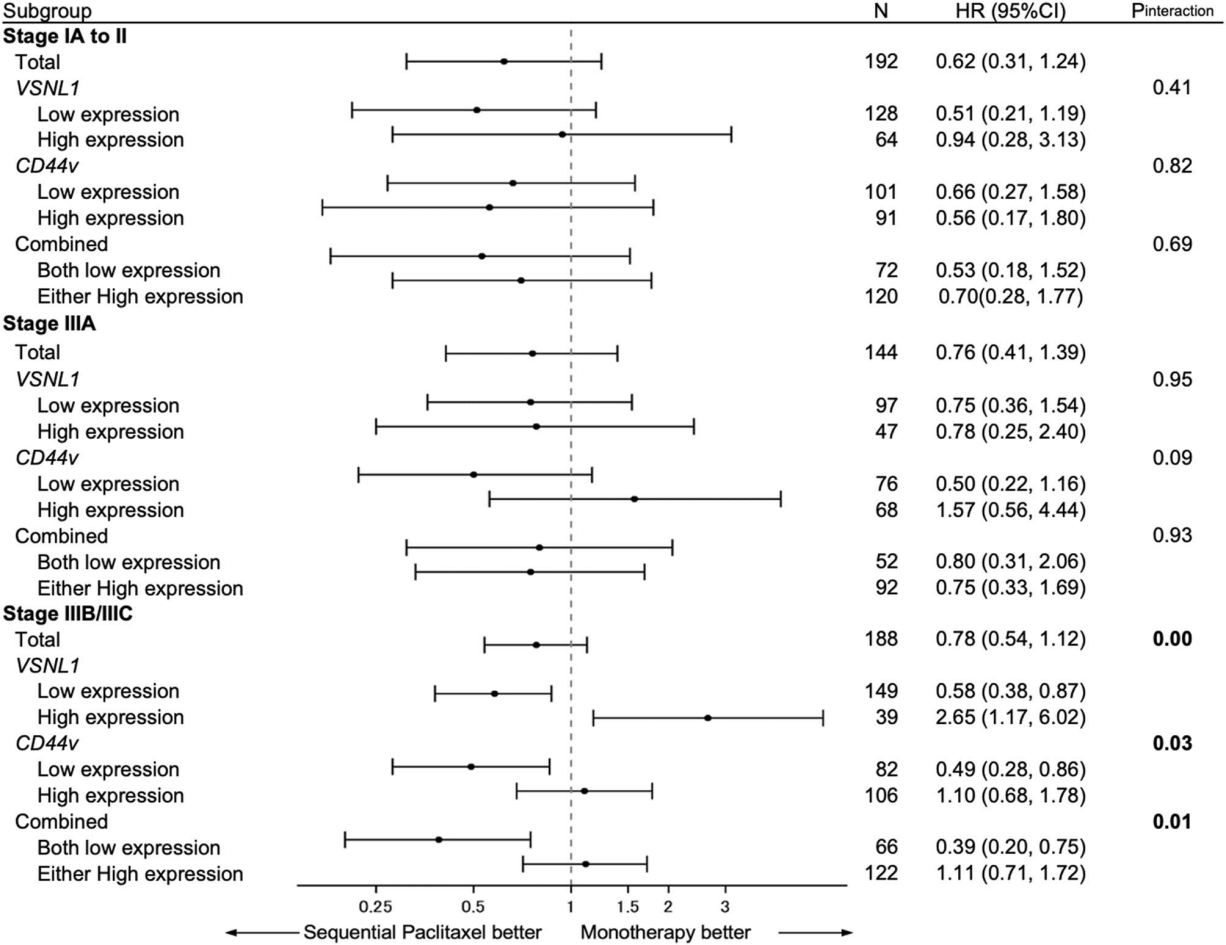
Forest plot of the study results. After patient stratification based on the pTNM stage, the survival benefit from sequential paclitaxel treatment was greater among patients with stage IIIB gastric cancer with a low expression of either gene or both. The association between the low expression levels of *VSNL1* and *CD44* and potential benefits from sequential paclitaxel treatment were significant for disease-free survival and cumulative incidence of relapse.

Patient stratification based on pTNM stage showed that OS improvement in response to sequential paclitaxel treatment in patients with low *VSNL1* and/or *CD44v* expression was the greatest in patients with stage IIIB/IIIC GC (Fig. 3).

### Internal validation

The overall performances of the different statistical models, including the interactions between *VSNL1* mRNA expression and the treatment group, as well as the clinical and pathological factors for OS prediction with C statistics using the bootstrap 0.632 + estimator (0.7111) and apparent estimator (0.7266), were evaluated. The accuracy of OS prediction based on *CD44* and *VSNL1* mRNA expression levels was comparable when the apparent estimator was used (0.7252), whereas it was not sufficiently accurate when the bootstrap 0.632 + estimator was used (0.7083) (Supplementary Table S3, Online Resource 1).

### Relationship between *VSNL1* or *CD44v* mRNA expression and clinicopathological factors

Significant relationships between the expression level of *VSLN1* mRNA and age, histopathological type, and pTNM stage were observed. Patients with low expression levels of VSNL1 mRNA in GC tissue had significantly higher rates of age < 65 years, undifferentiated adenocarcinoma, and high pTMN stage compared to those with high expression. In contrast, there was no significant relationship between *CD44* mRNA expression and any clinicopathological factors (Supplementary Table S4, Online Resource 1).

### Relationship between mRNA expression levels and protein expression levels of *VSNL1* and* CD44v*

Protein expression levels of VSNL1 and CD44 were investigated in a subgroup of patients based on immunohistochemistry (IHC) analyses, and patients were dichotomized into low and high expression groups, based on an immune response scoring system.

**Figure 4:**
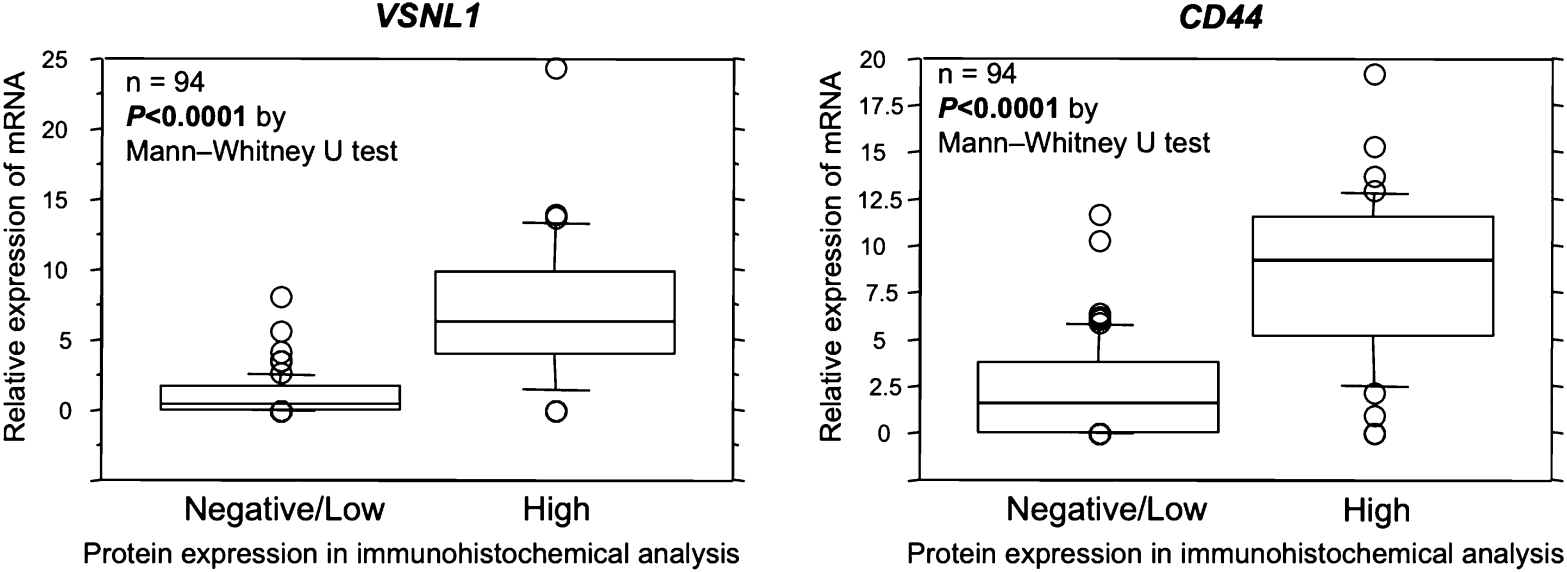
Relationship between mRNA expression levels and protein expression levels of VSNL1 and CD44. The protein expression levels of VSNL1 and CD44 based on immunohistochemistry (IHC) analysis were divided into low and high expression groups. There was a significant difference in the mRNA expression levels of VSNL1 and CD44 between the low and high protein expression groups of both VSNL1 and CD44 based on IHC analysis**.**

For CD44v IHC, since there are eight variant isoforms (CD44v1-8) created by mRNA splice variants, we analyzed the relationship between CD44v1-8 and CD44 using data from NanoString analysis and found that all CD44v mRNA expression was strongly correlated with that of CD44 mRNA (Supplementary Fig. S1, Online Resource 1). Therefore, CD44 expression in IHC was examined as a representative of CD44 and CD44v1-8. The relationship between VSNL1 and CD44 protein expression levels and mRNA expression levels by IHC analysis showed that mRNA expression levels were significantly higher in the high protein-expression group than in the low-protein expression group, based on the Mann–Whitney U test (Fig. 4; P < 0.0001, P < 0.0001, respectively). In addition, the concordance between high/low mRNA expression levels and high/low protein expression levels were 79.8% and 81.9% for VSNL1 and CD44, respectively (Table 3).

**Table 3:** Relationship between VSNL1 mRNA expression and VSNL1 protein expression, and for the relationship between CD44 mRNA expression and CD44 protein expression.

	VSLN1				CD44		
	IHC high	IHC low			IHC high	IHC low	
mRNA high	27	13	40	mRNA High	31	11	42
mRNA low	6	48	54	mRNA Low	6	46	52
	33	61			37	57	
Concordance rate: 79.8%				Concordance rate: 81.9%			

Furthermore, patients were divided into low expression groups of both VSNL1 and CD44 proteins (n = 53) and high expression groups of either VSNL1 or CD44 protein (n = 41), according to the VSNL1 and CD44 protein expression results in the IHC analyses. In each group, the OS of sequential paclitaxel and fluoropyrimidine monotherapy was evaluated using a log-rank test. The results showed that the OS of sequential paclitaxel was significantly better than that of fluoropyrimidine monotherapy in patients with low levels of expression of both VSNL1 and CD44. Conversely, no difference was observed in the high expression groups of either VSNL1 or CD44 (Fig. 4), which was consistent with the mRNA results.

### Examination of the usefulness of the algorithm with the four biomarkers (GZMB, WARS, SFRP4, and CDX1) validated in the CLASSIC study sample to stratify the risk of recurrence and select patients who would benefit from adjuvant chemotherapy with paclitaxel followed by sequential pyrimidine fluoride using the sample from this biomarker study

In the sample of the current biomarker study (n = 527), the algorithm based on GZMB, WARS, and SFRP4 mRNA expression levels did not significantly stratify the risk of recurrence (Supplementary Fig. 2a,b, Online Resource 1). Subsequently, when the patients were separated into "chemotherapy benefit group" and "chemotherapy no-benefit group" according to the algorithm based on GZMB, WARS, and CDX1 mRNA expression levels, in the chemotherapy no-benefit group, the survival rates of patients in the chemotherapy-responsive group were the same regardless of the type of adjuvant treatment. However, in the chemotherapy-naive group, characterized by high immunity (GZMB + , WARS +) and low epitheliotropism (CDX1-), patients treated with sequential paclitaxel had significantly longer survival (Supplementary Fig. 2c,d, Online Resource 1).

## Discussion

The present study explored biomarkers for identifying gastric cancer (GC) patients that are likely to benefit from sequential paclitaxel treatment followed by fluorinated-pyrimidine-based adjuvant chemotherapy at the mRNA level using clinical samples and data from GC patients treated in a randomized controlled phase III trial of adjuvant chemotherapy, SAMIT^15^. Although previous studies using clinical samples from the ACTS-GC have revealed several novel molecular GC biomarkers, significant interactions between S-1 treatment and RNA expression levels have not been observed^8–11^. In a study of clinical samples from the CLASSIC trial, an algorithm based on the RNA expression levels of three genes was able to predict patients who were likely to benefit from adjuvant chemotherapy with capecitabine plus oxaliplatin^12^.

Although several candidate biomarkers of resistance or sensitivity to paclitaxel, such as Tau, COL4A3BP, UGCG, MCL1, FBW7, SLC31A2, SLC35A5, SLC43A1, SLC41A2, and CCNG1 have previously been suggested^16–23^, none have been validated in a second independent series. Hence, there remains a clinical need to validate the proposed biomarkers and/or identify new biomarkers that can be used in routine clinical practice to identify patients likely to benefit from paclitaxel therapy^24^. Moreover, associations between the expression of several genes or proteins and the benefits of paclitaxel, such as CCND1, ABCB1, BCL-2, and SPARC in different tumor types, have been reported in multiple studies ^25–29^. For example, CCND1 overexpression promotes paclitaxel-induced apoptosis in breast cancer^26^. BCL-2 family members such as BCL-2, BCl-xL, BAX, and ABCB1, have been reported to be involved in paclitaxel resistance in esophageal cancer^27^. In addition, SPARC expression in tumor stromal cells is a potential negative predictor of paclitaxel treatment in patients with lung cancer^28,29^. However, the expression levels of all previously suggested biomarkers were not significantly associated with patient outcomes in the present study. This may be related to the cancer type, sample size, case mix, ethnic differences, or methodological differences.

In the present study, we identified the expression levels of VSNL1 and/or CD44v as potential novel predictive biomarkers to identify patients who could benefit from postoperative adjuvant chemotherapy with sequential paclitaxel followed by a fluorinated-pyrimidine after curative gastrectomy. Although the combined low expression of the two biomarkers predicted the greatest benefits from adjuvant chemotherapy with sequential paclitaxel and a fluorinated-pyrimidine, no clear interaction between VSNL1 and CD44v has been reported to date.

The *VSNL1* gene encodes visinin-like protein 1 (VILIP-1), a member of the neuronal calcium sensor protein family that regulates calcium-dependent cells and signaling adenylate cyclase^30^. In cancers, VSNL1 is overexpressed in various cancers such as GC, colorectal cancer, non-small cell lung cancer, and squamous cell carcinoma^31–34^, and inhibits cell proliferation, adhesion, and infiltration. In addition, it has been reported to function as a tumor suppressor gene^33,34^. Deficiency or reduced expression of VSNL1 by knockdown in vitro has been reported to increase the motility of cancer cells, suggesting a potential tumor suppressor function of the protein. VSNL1 regulates SNAIL1, which is a transcription factor with cAMP-dependent function, and SNAIL1 expression prevents epithelial-mesenchymal transition in cancer cells^34^. In recent years, it has been reported that high expression of VSNL1 promotes the proliferation and migration of GC cells by regulating the expression of P2X3 and P2Y2 receptors, and that high expression of VSNL1 in GC tissue may be a good clinical indicator for poor prognosis in GC patients^35^. However, in the present study, VSNL1 expression in GC tissue was not a prognostic factor. Regarding the association with chemotherapy, VSNL-1 has been reported to be involved in epithelial-mesenchymal transition (EMT) of cancer cells by regulating the transcription factor Snail1 in a cAMP-dependent manner ^34^. Therefore, high expression of VSLN1 suppresses EMT by regulating Snail1, which may weaken chemoresistance to anticancer agents, including paclitaxel, and increase chemosensitivity.

The *CD44* gene encodes the CD44 protein, an adhesion molecule that uses hyaluronan as a ligand, and there are eight isoforms (CD44v1-8) that are created by mRNA splice variants. In the present study, we initially investigated only *CD44v1* mRNA expression and identified it as a biomarker. Additional analysis of the relationship between *CD44v1-8* and *CD44* using data from NanoString analysis showed that the expression of all *CD44v* isoforms was strongly correlated with *CD44* expression, indicating that *CD44* and *CD44v1-8* mRNA expression may be biomarkers in the present study. CD44 protein is overexpressed on the cell surface of cancer stem cells in GC tissues, and binding of hyaluronan to CD44 has been reported to affect various downstream signaling pathways, leading to cancer invasion, metastasis, and resistance to chemoradiotherapy^36–42^. As for paclitaxel resistance, ovarian cancer has been reported to exhibit higher levels of CD44 expression than paclitaxel-sensitive cancer cells^43^.

To the best of our knowledge, this is the first and most comprehensive study to identify biomarkers for the prediction of patients with survival benefit from sequential paclitaxel followed by fluorinated-pyrimidine adjuvant chemotherapy in GC patients. However, the present study has several limitations. First, although we demonstrated that the study cohort was representative of the entire SAMIT patient cohort, with respect to clinicopathological characteristics, including survival, we were only able to retrieve material from approximately a third of the original SAMIT population. Furthermore, the number of samples in which biomarkers identified at the mRNA level were validated at the protein level was limited. Second, we only analyzed RNA samples from a single tissue block, not whole tumors. Therefore, the intertumoral heterogeneity may not be sufficiently assessed. Third, SAMIT recruited patients with serosal invasion (e.g., cT4 tumors), a major risk for peritoneal recurrence, and randomized them to receive fluorinated pyrimidine monotherapy or sequential paclitaxel, which was hypothesized to reduce postoperative recurrence, such as peritoneal recurrence, and improve prognosis. However, it should be noted that there was a small number of patients with pT4 tumors in the SAMIT.

In conclusion, the biomarkers for selecting patients with GC who would most likely benefit from adjuvant chemotherapy with sequential paclitaxel and fluorinated-pyrimidine treatment after curative gastrectomy were identified. Although the validation of our findings in a second independent series followed by a prospective trial is necessary, personalized adjuvant chemotherapy using these biomarkers may further improve treatment outcomes in patients with locally advanced GC.

## Methods

### Patients and sample collection

This biomarker study was conducted using GC specimens and clinicopathological data from patients who participated in a phase 3 randomized comparative study (SAMIT) performed using a two × two factorial design of postoperative adjuvant chemotherapy after D2 gastrectomy. SAMIT was performed in 230 hospitals in Japan in patients with GC. Patients aged 20–80 years with an ECOG performance score of 0–1 who were diagnosed with cT4a or T4b GC by preoperative diagnosis were enrolled. The patients were randomly assigned to one of the four postoperative adjuvant chemotherapy groups (tegafur and uracil [UFT] monotherapy, S-1 monotherapy, three courses of paclitaxel followed by UFT, or three courses of paclitaxel followed by S-1) after undergoing D2 gastrectomy.

The completion rate of the trial was 60% in the UFT-only group, 62% in the S-1-only group, 68% in the UFT-treated group after paclitaxel treatment, and 70% in the S-1-treated group after paclitaxel^15^.

The present study was approved by the Institutional Review Board (IRB) of Kanagawa Cancer Center, the central institute for this study (approval number: 26-42), as well as the IRBs of all institutions that participated in the present study. Representative blocks from formalin-fixed paraffin-embedded (FFPE) gastrectomy specimens were collected retrospectively from participating institutions according to the following inclusion criteria: (1) patients were participants in the SAMIT, (2) FFPE blocks or unstained cut sections were available, and (3) the translational study protocol was approved by the IRB. Samples were collected from the data center of the Kanagawa Cancer Center and shipped to Yokohama City University for RNA extraction and analysis. Sections (each 10-μm thick) were cut from the FFPE blocks and stored at 4 °C until microdissection.

### RNA extraction and complementary DNA (cDNA) synthesis

Hematoxylin and eosin-stained slides were reviewed, and the area with the highest tumor content was manually outlined. After manual microdissection, total RNA was isolated using NucleoSpin FFPE RNA XS (Macherey-Nagel GMBH & Co. KG, Düren, Germany). For RNA quality control, the OD_260_/OD_280_ ratio was measured using a NanoDrop 2000 (Thermo Fisher Scientific Inc., MA, USA; RRID:SCR_018042). The total RNA integrity number was measured using an Agilent 2100 Bioanalyzer (Agilent Technologies Inc., Waldbronn, Germany, RRID:SCR_018043). To confirm that the total RNA samples were not contaminated with DNA, *RNA18S1* expression was evaluated by quantitative real-time PCR (qRT-PCR) in each sample before cDNA preparation. cDNA was prepared from samples that passed all the quality control checks. cDNA was synthesized from 0.4 µg of total RNA using an iScript cDNA Synthesis Kit (BIO-RAD LABORATORIES, Inc., CA, USA), diluted to 0.2 µg/µL with distilled water, and stored at − 20 °C until use.

### qRT-PCR

qRT-PCR was performed using the QuantiFast Probe Assay (Qiagen, Venlo, Netherlands) and a QuantiFast Probe PCR (Qiagen) according to the manufacturer’s instructions. The expression of each gene was quantified in triplicate. A standard curve was plotted for each run using three fixed concentrations of human control cDNA synthesized using Xpress Ref Universal Total RNA (Qiagen) with an iScript cDNA Synthesis Kit (Bio-Rad Laboratories, Inc.) to measure the mRNA expression levels in all samples. The concentration of each sample was determined based on the point of intersection of the sample value with the standard curve. *β-actin* and *RNA18S1* were used as the internal controls.

### Gene selection

The RNA expression levels of 105 genes were quantified in the present study (Table 4). Fifty-eight genes were selected from a previous DNA microarray study^44^. An additional 47 genes were selected from 14 categories previously linked to tumor progression or survival in GC patients, along with 14 genes that did not overlap with the 58 genes mentioned above. The 14 categories are described in Table 4 (categories 1–14).

**Table 4:** Genes investigated (n = 105).

**1. Genes encoding proteins related to the metabolism or activation of anticancer agents**							
*TYMS*	*DPYD*	*UMPS*	*UPP1*	*TYMP*	*GGH*	*DUT*	*MTHFR*
*RRM1*	*RRM2*	*FPGS*	*DHFR*	*TOP1*	*ERCC1*	*TOP2A*	*MAPT*
**2. Genes encoding growth factors and receptor tyrosine kinases**							
*EGF*	*AREG*	*EREG*	*VEGFA*	*IGF2*	*HGF*	*MET*	*FGFR2*
*EGFR*	*ERBB2*	*ERBB3*	*KDR*	*IGF1R*	*PDGFRB*		
**3. Genes encoding proteins related to the p13K-AKT, RAS, and RAP1 signaling pathways**							
*PIK3CA*	*JAK2*	*PTEN*	*ITGB3*	*PLA2G2A*	*THBS1*		
**4. Tumor suppressor protein-encoding genes**							
*SEMA3B*	*RUNX3*	*MLH1*	*APC*	*DAPK1*	*MGMT*	*CDKN2A*	
**5. Genes encoding apoptosis-related proteins**							
*E2F1*	*BCL-2*	*GADD45*	*FAS*	*BIRC5*	*BCL-xL*	*BAX*	*CCND1*
**6. Genes related to cancer stem cells**							
*LGR5*	*PROM1*	*CD44v*	*NANOG*	*MSI1*			
**7. Genes related to anticancer drug resistance**							
*ABCG2*	*ABCB1*	*ABCC1*	*CAV1*				
**8. Genes encoding members of the MMP family**							
*MMP2*	*MMP7*	*MMP9*	*MMP10*	*MMP11*	*MMP14*	*TIMP1*	
**9. Genes encoding cell adhesion factor and ECM**							
*CDH17*	*LGALS4*	*VCAM1*	*HPSE*	*DSG2*	*CDX2*		
**10. Genes encoding members of the claudin family**							
*CLDN3*	*CLDN4*	*CLDN7*	*CLDN18.2*				
**11. Genes encoding chemokine receptors**							
*CCR7*	*CXCR4*						
**12. Genes related to immune checkpoint regulation**							
*PDL1*	*PDL2*						
**13. Epigenetic repression genes**							
*HDAC1*	*EZH2*						
**14. Genes identified by SAGE and CAST methods**^17^							
*APOE*	*REG4*	*MIA*	*OLFM4*	*SEC11A*	*TSPAN8*	*TM9SF3*	*ZDHHC14*
**15. Other genes**							
*INHBA*	*LDHA*	*PTGS2*	*VSNL1*	*TGFA*	*MUC13*	*SIRT1*	*GZMA*
*ESR1*	*MUC2*	*SPARC*	*ANGPT2*	*PLAU*	*PECAM1*		

The 105 selected genes included 63 genes analyzed in an exploratory biomarker study of ACTS-GC participants^10^. Among them, 57 genes have been previously reported as biomarkers of paclitaxel resistance or sensitivity. The functional annotation of each gene carried out using DAVID 6.7 (https://david-d.ncifcrf.gov/), is outlined in Supplementary Table S6 (Online Resource 1).

### Defining the predictive value of the biomarkers

The mRNA expression level of each gene was classified as low versus high using the median mRNA expression level as a cut-off point, as described previously^44^. If the mRNA expression level of a particular gene was below 1.0 × 10^–8^ ng/μL, the expression level was set to ‘0.00’. The value of a biomarker in predicting the benefit of sequential paclitaxel treatment based on the OS, DFS, and cumulative incidence of relapse was determined by examining the *p*-values of the interactions between the dichotomized gene expression level and the treatment group (sequential paclitaxel versus monotherapy) after adjusting for clinical and pathological factors using Cox regression or Fine-Gray models^45,46^. The genes were ranked according to treatment interaction-related *p*-values. Values were considered significant at *p* < 0.05. Additionally, we combined the expression levels of selected genes to identify sensitive and non-sensitive patient subsets.

### Immunohistochemistry (IHC) of VSLN1 and CD44

IHC of VSLN1 and CD44v was performed using FFPE specimens from 94 patients who participated in SAMIT at the Kanagawa Cancer Center. Tissue sections were deparaffinized and incubated in 10 mM sodium citrate buffer (pH 6.0) at 121 °C for 15 min for antigen retrieval. Sections were incubated with primary antibodies overnight at 4 °C. Anti-VSNL1 (UM870034; ORI GENE TECHNOLOGIES, Inc. MD, USA, diluted at 1:200 with PBS [pH 7.3] containing 1% BSA, 50% glycerol, and 0.02% sodium azide) and anti-CD44 (ab51037; ABCAM PLC, Cambridge, UK, dilution at 1:100 with PBS [pH 7.3] containing 1% BSA, 50% glycerol, and 0.02% sodium azide) were used. Preliminary testing was performed using positive controls to determine the optimal dilution of each antibody. Peroxidase-labeled polymers (EnVision + , Rabbit, DAKO, Glostrup, Denmark) and diaminobenzidine were used for detection. All sections were counterstained with hematoxylin. Immunohistochemical assessments were performed based on the Immune Response Scoring system. Intensity scores were used to classify the strongest positive immunostaining tumor cells as absent (score 0), weak (score 1), moderate (score 2), and strong (score 3). Typical VSNL1 and CD44 intensity score classifications are shown in Supplementary figures S3a, b. Proportion scores were used to classify the proportions of positive immunostained tumor cells into four grades (0, 1, 2, 3, 4, and 5) based on a marker-specific approach (Supplementary Fig. S4). The sum of the scores for the intensity and proportion scores ranges from 0 to 8. A score of 0–4 was defined as negative/low protein expression, and a score of 5–8 was defined as high protein expression, in both VSNL1 and CD44.

### Examination of the relationship between VSNL1 and CD44 mRNA expression and those protein expression

We investigated each VSNL1 and CD44 mRNA expression levels in each negative/low protein expression group or high protein expression group. In addition, we investigated the concordance between mRNA expression levels split into two by the median used in the present study and the protein expression levels in immunohistochemical analyses. In addition, patients were divided into low expression groups for both VSNL1 and CD44 and high expression groups of either VSNL1 or CD44, according to VSNL1 and CD44 protein expression in IHC. In each group, the OS of sequential paclitaxel and fluoropyrimidine monotherapy was evaluated.

### Internal validation

We adopted an internal validation strategy, as proposed by Wahl et al.^47^, to address the potential overestimation of the standard error owing to multiple imputations and optimism in the predictive performance. We used Harrell’s C statistics to analyze the predictive performance of the survival data and addressed the optimistic bias by Harrell’s C statistics using the bootstrap 0.632 + method with 20 bootstrap samples from the original dataset with replacement, followed by multiple imputations.

### Statistical analysis

The pre-defined statistical analysis plan for this study has been reported previously^48^. The primary and secondary endpoints were the OS and DFS, respectively. The OS and DFS curves were constructed using the Kaplan–Meier method, and the cumulative incidence curves of relapse were constructed using the Aalen-Johansen method^49^ to compare sequential paclitaxel and monotherapy, considering the expression levels of the selected genes either individually or in combination. The adjusted hazard ratios (HRs), 95% confidence intervals (CIs), and *p*-values of the major treatment effects and interactions were estimated for the entire patient population and subgroups according to the Union for International Cancer Control TNM 8th ed stage^2^. We used multiple imputations to handle missing clinical and pathological factor data and generated 20 multiply imputed datasets for parameter estimates. The reported *p*-values were two-tailed, and the major effects and interactions were considered statistically significant at *p* < 0.05. Statistical analyses were performed using SAS version 9.4 (SAS INSTITUTE, Inc., Cary, NC, USA).

### Ethical statement

All procedures followed were in accordance with the ethical standards of the responsible committee on human experimentation (institutional and national) and with the Helsinki Declaration of 1964 and later versions. Informed consent or a substitute for it was obtained from all patients for inclusion in the study.

## Supplementary Information

Supplementary Figure S1.

Supplementary Figure S2.

Supplementary Figure S2.

Supplementary Figure S2.

Supplementary Figure S2.

Supplementary Figure S3.

Supplementary Figure S3.

Supplementary Figure S4.

Supplementary Table S1.

Supplementary Table S2.

Supplementary Table S3.

Supplementary Table S4.

Supplementary Table S5.

Supplementary Table S6.
