# Supplementary figures and images for: Gastric cancer biomarker analysis in patients treated with different adjuvant chemotherapy regimens within SAMIT, a phase III randomized controlled trial
Source: Sci Rep. 2022 May 20;12:8509. doi: 10.1038/s41598-022-12439-3 (PMC9123164; doi:10.1038/s41598-022-12439-3)

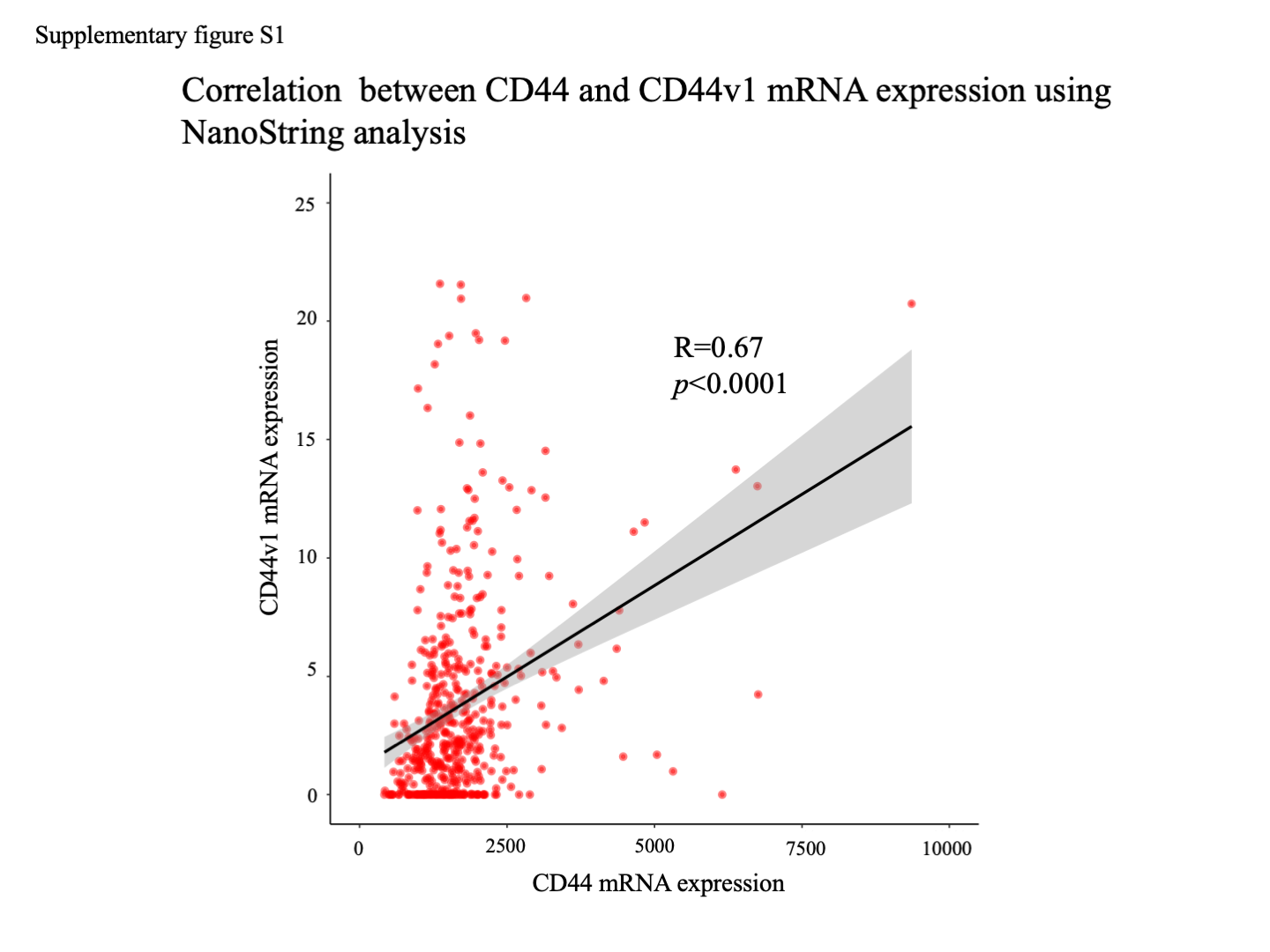

Supplement: Supplementary file 1 — Supplementary Figure S1. [file 41598_2022_12439_MOESM1_ESM.tiff]

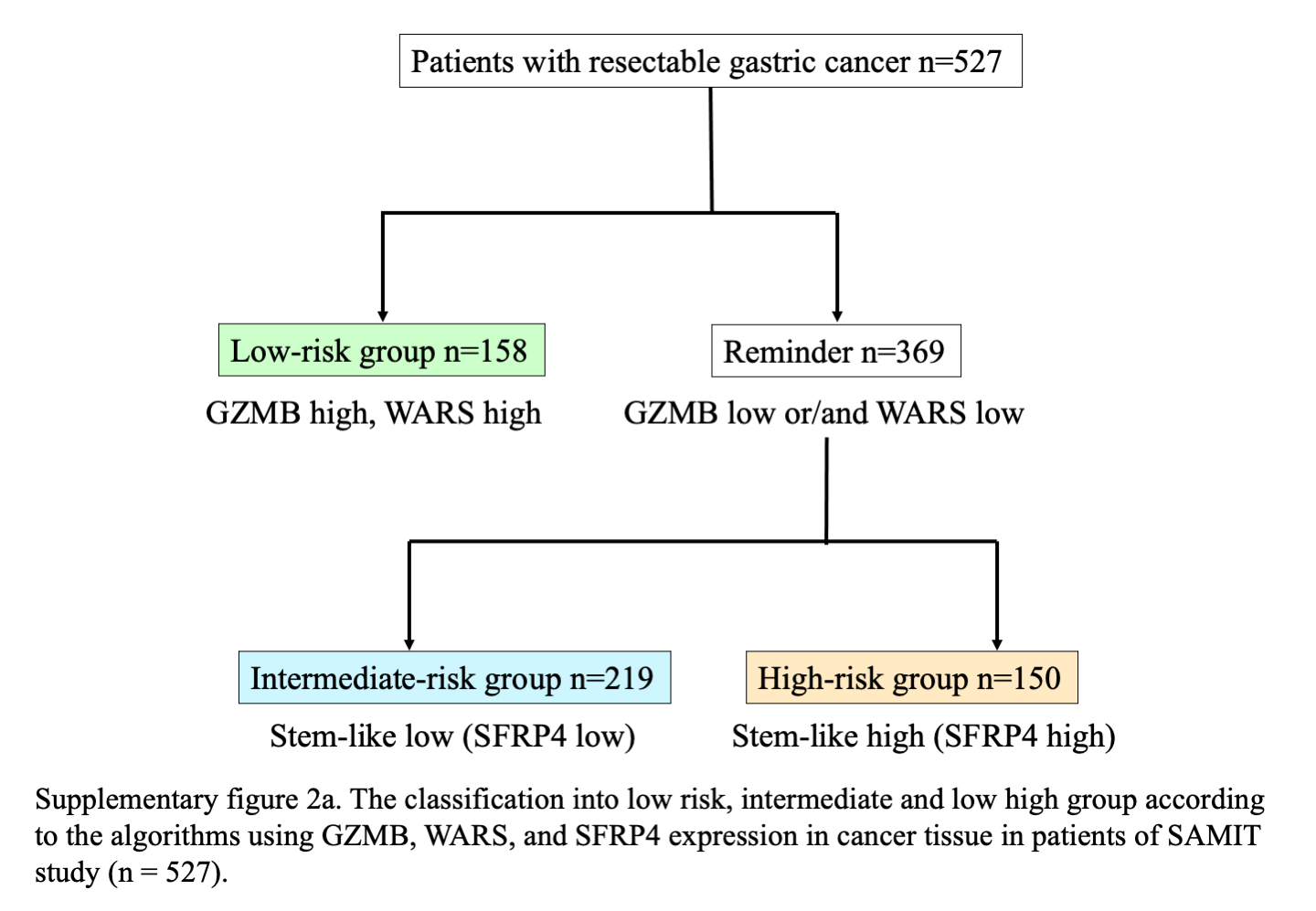

Supplement: Supplementary file 2 — Supplementary Figure S2. [file 41598_2022_12439_MOESM2_ESM.tiff]

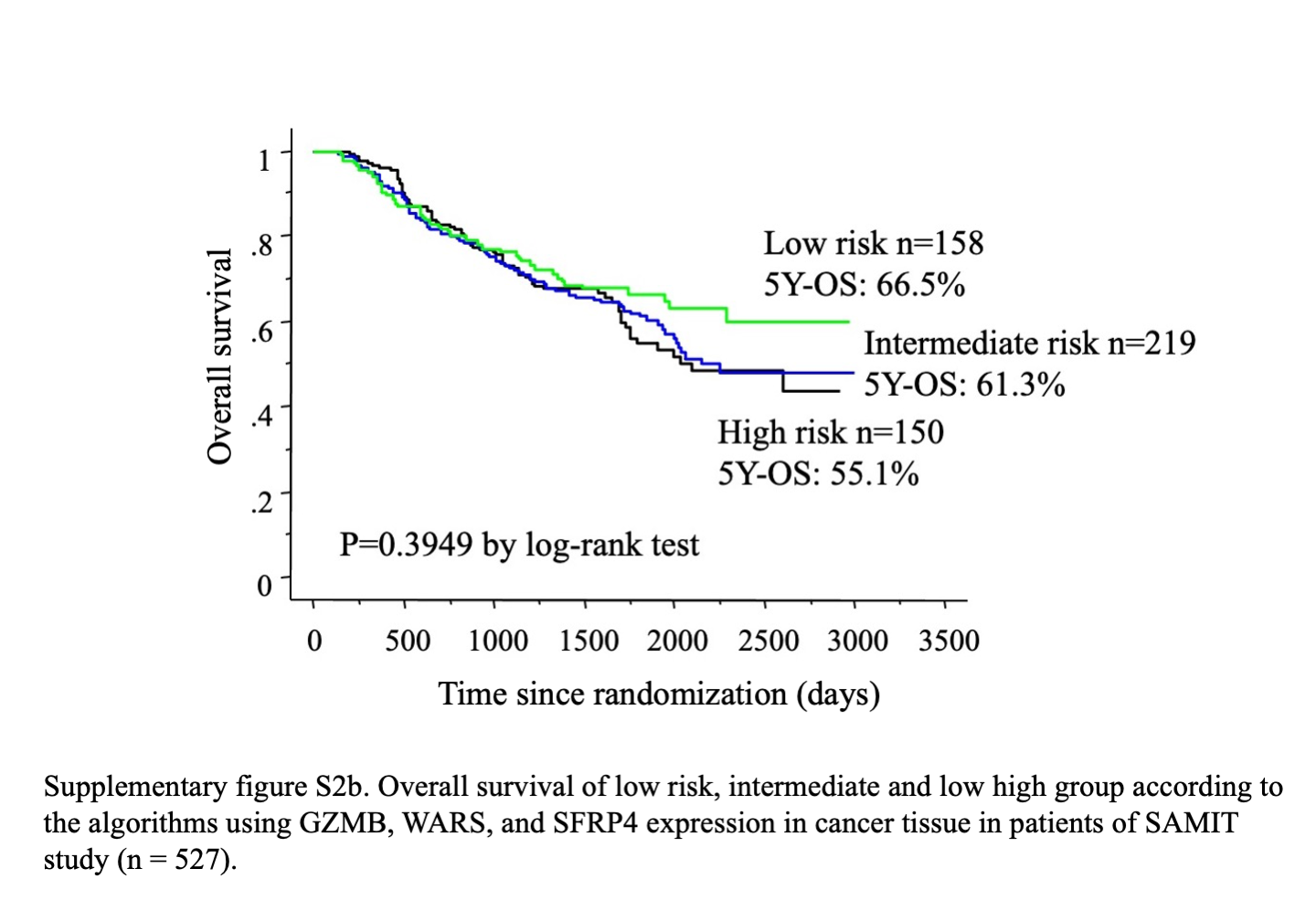

Supplement: Supplementary file 3 — Supplementary Figure S2. [file 41598_2022_12439_MOESM3_ESM.tiff]

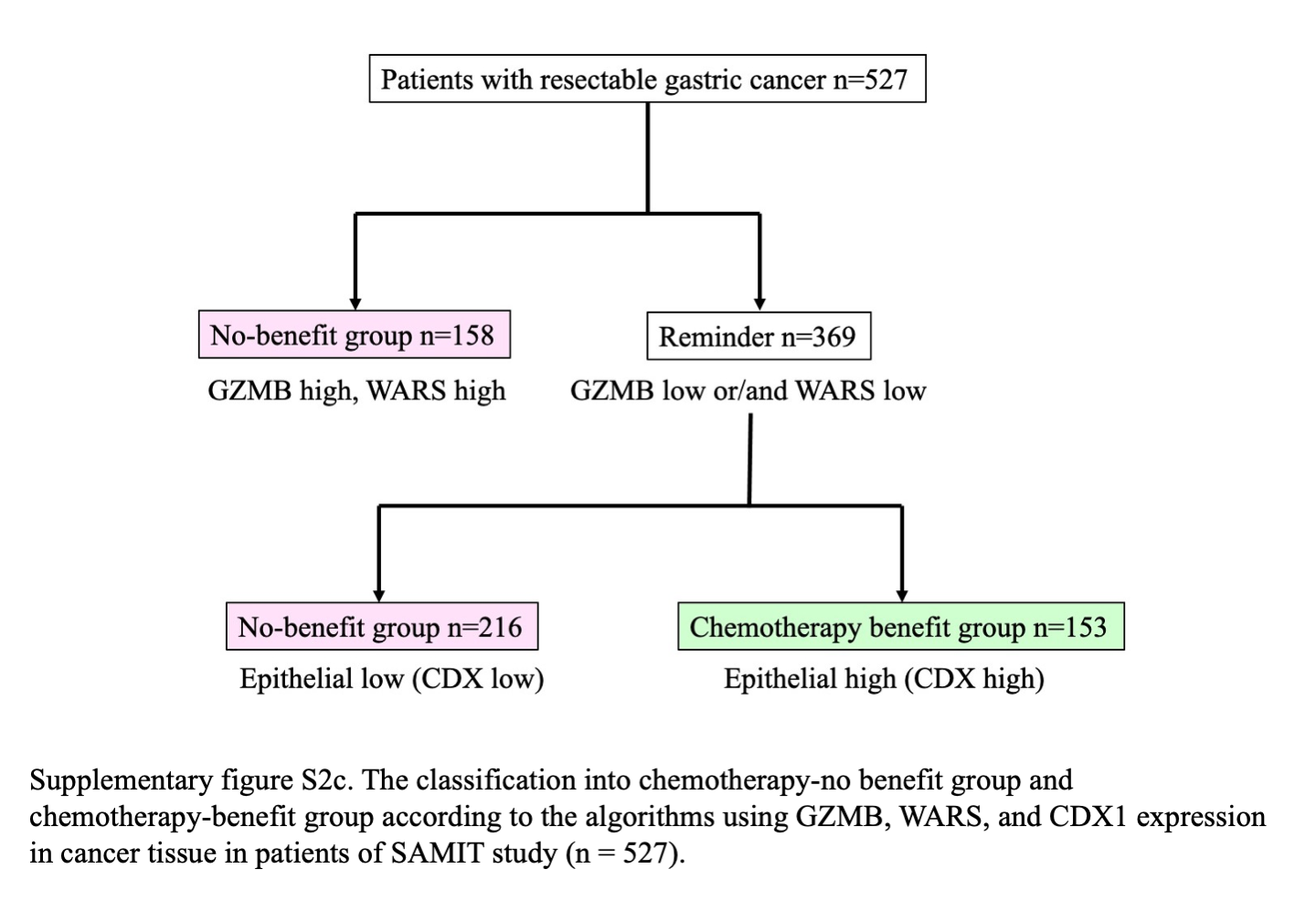

Supplement: Supplementary file 4 — Supplementary Figure S2. [file 41598_2022_12439_MOESM4_ESM.tiff]

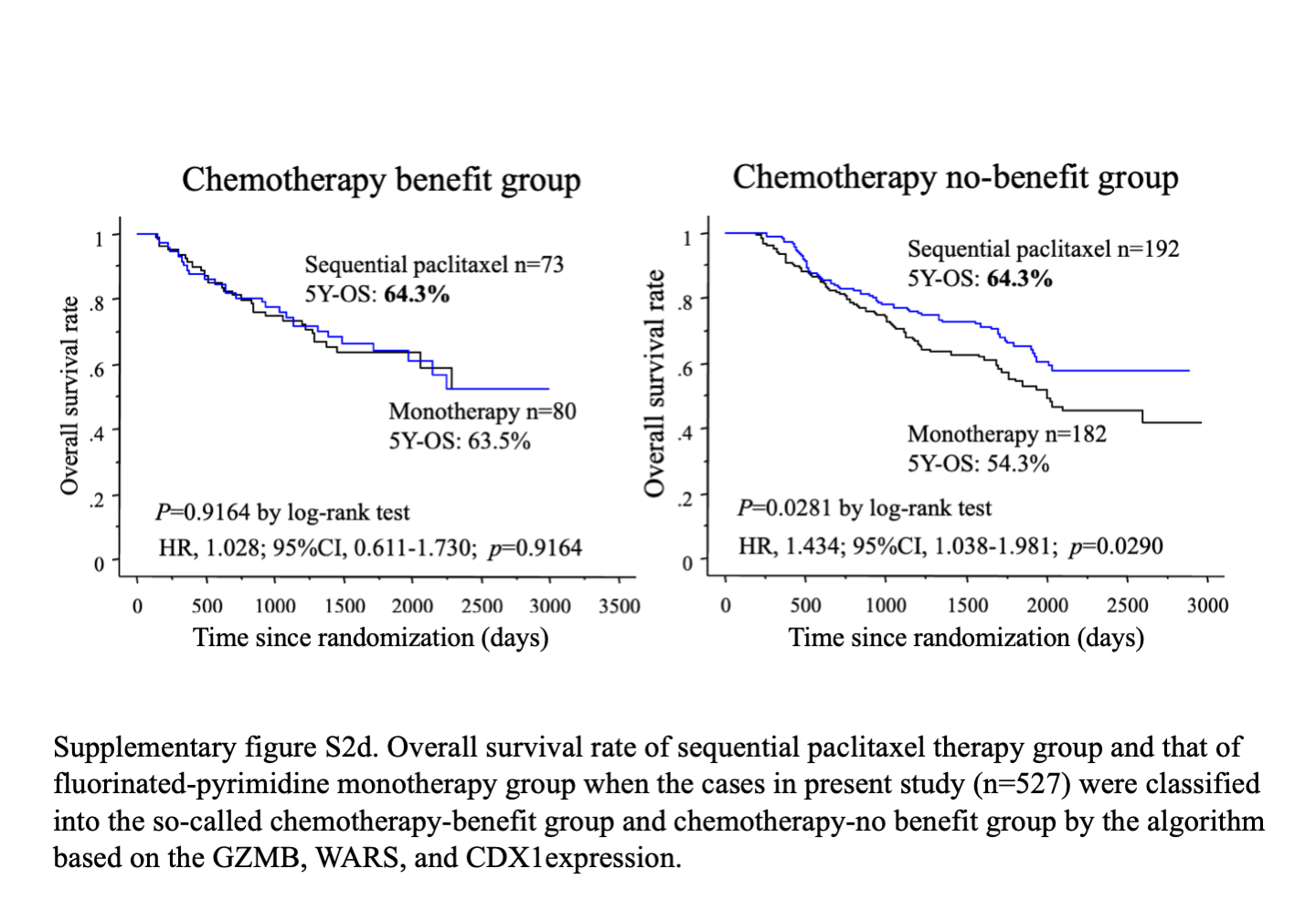

Supplement: Supplementary file 5 — Supplementary Figure S2. [file 41598_2022_12439_MOESM5_ESM.tiff]

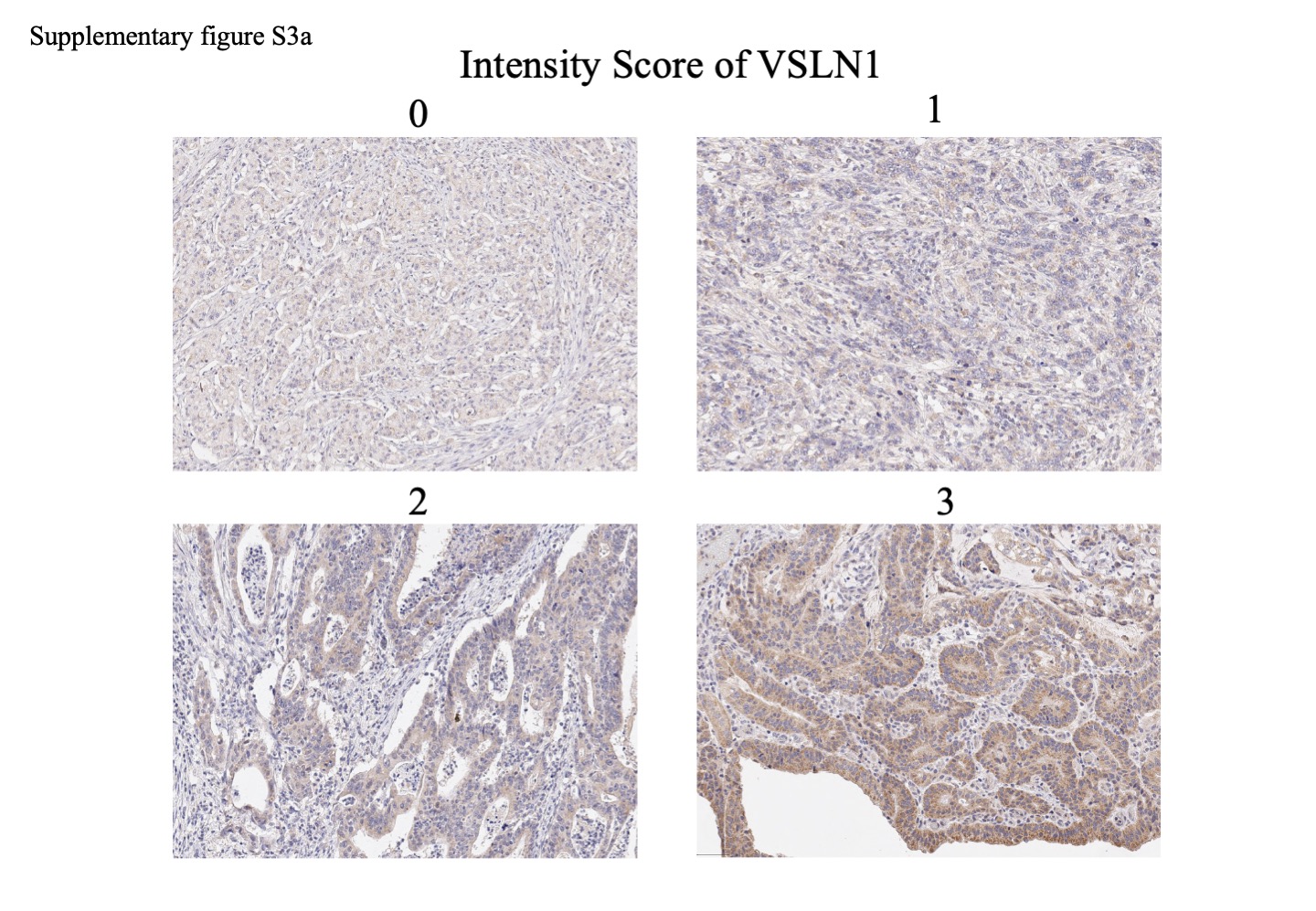

Supplement: Supplementary file 6 — Supplementary Figure S3. [file 41598_2022_12439_MOESM6_ESM.jpeg]

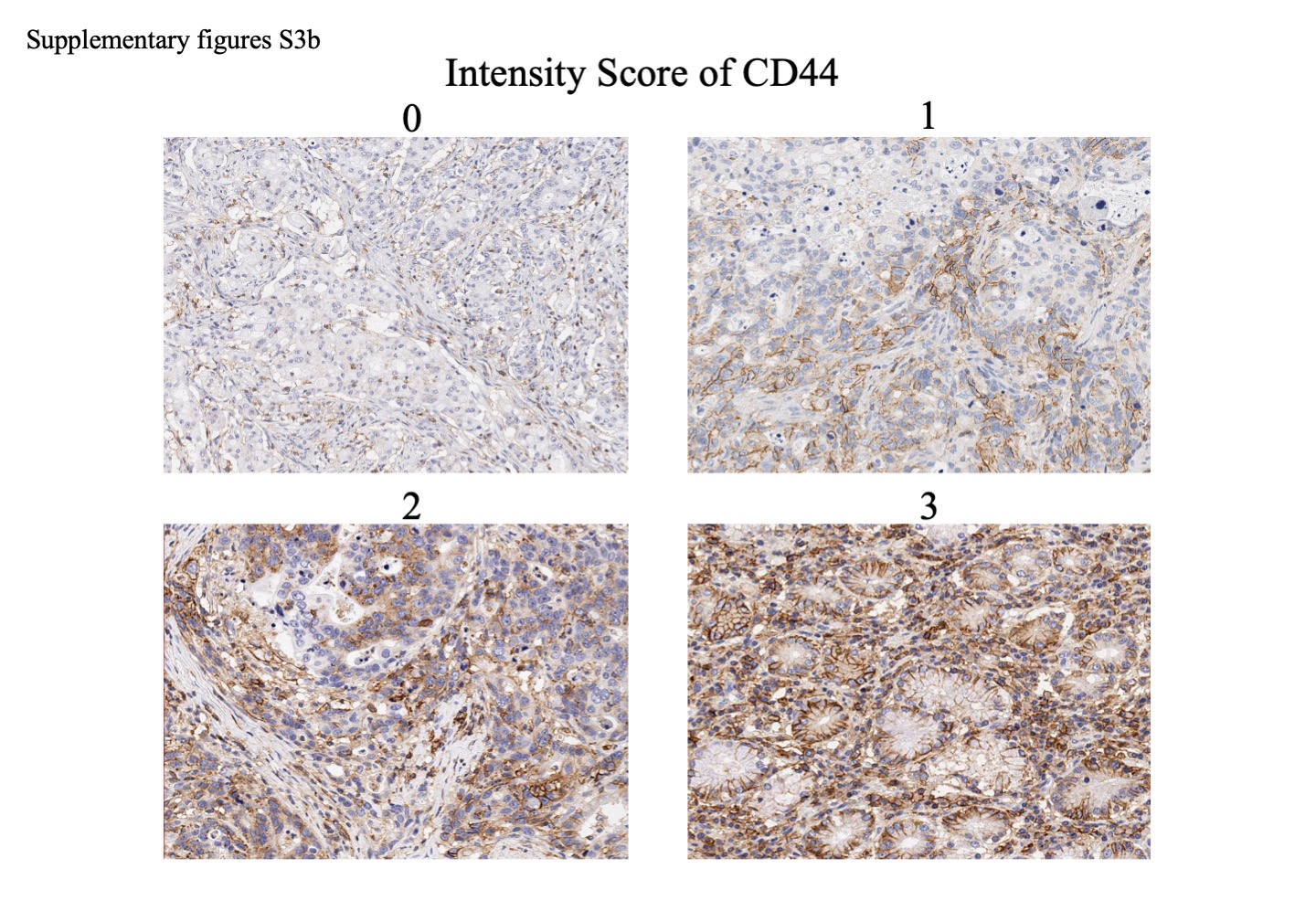

Supplement: Supplementary file 7 — Supplementary Figure S3. [file 41598_2022_12439_MOESM7_ESM.jpeg]

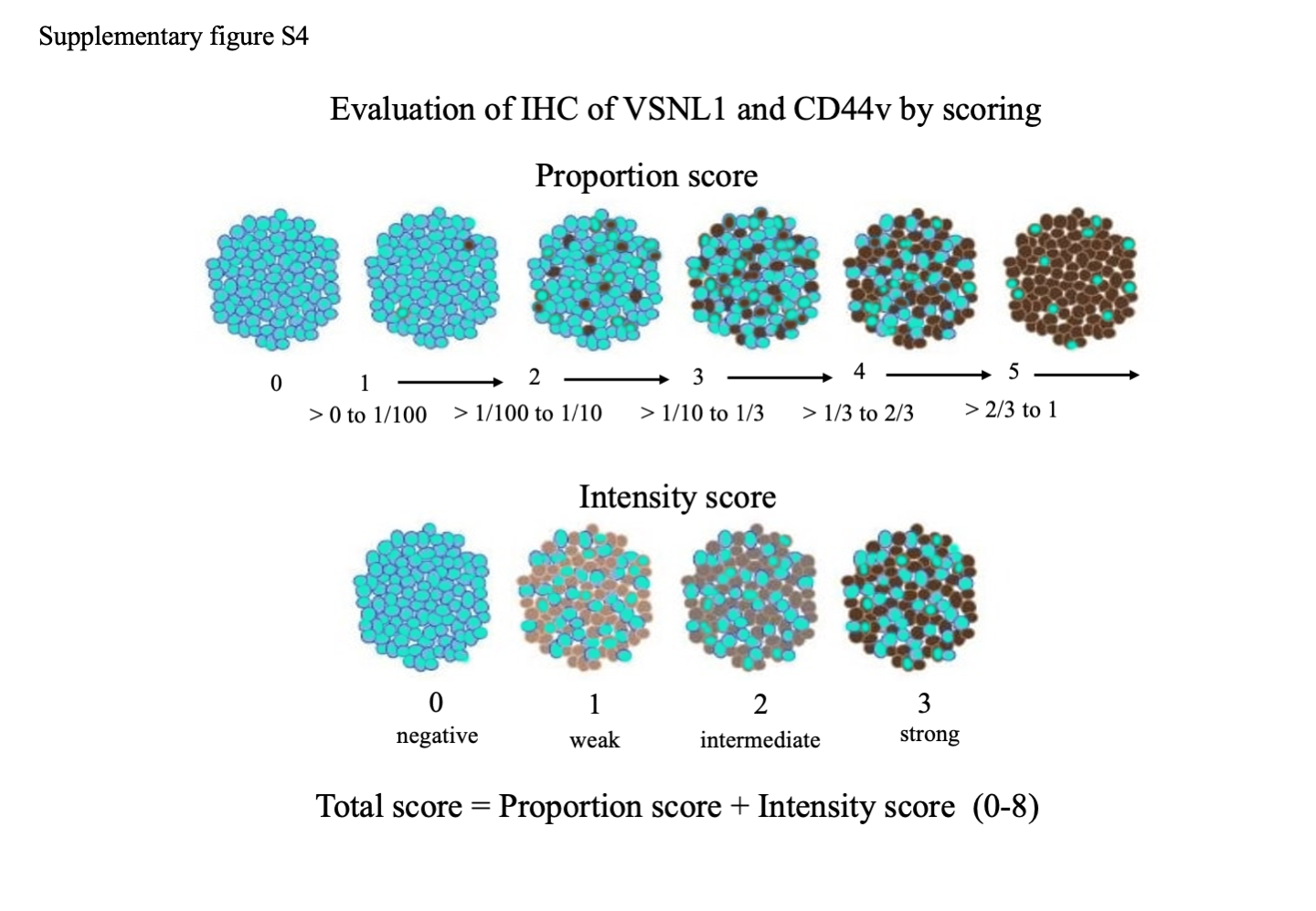

Supplement: Supplementary file 8 — Supplementary Figure S4. [file 41598_2022_12439_MOESM8_ESM.tif]
