# Supplementary material for: Gastric cancer biomarker analysis in patients treated with different adjuvant chemotherapy regimens within SAMIT, a phase III randomized controlled trial
Source: Sci Rep. 2022 May 20;12:8509. doi: 10.1038/s41598-022-12439-3 (PMC9123164; doi:10.1038/s41598-022-12439-3)
Supplement: Supplementary file 9 — Supplementary Table S1. [file 41598_2022_12439_MOESM9_ESM.docx]

| **Supplementary table S1.** Clinical and pathological characteristics of 527 patients with gastric cancer administered monotherapy or sequential paxclitaxel, with gene expression data segregated according to the treatment arms | | | | | | | | |
| --- | --- | --- | --- | --- | --- | --- | --- | --- |
|  |  | Monotherapy  (*n*=262) | |  | Sequential paclitaxel (*n*=265) | |  |  |
|  |  | No. of patients | Proportion |  | No. of patients | Proportion |  | *p*-Value |
| Arms |  |  |  |  |  |  |  |  |
|  | S-1 only | 128 | 48.9% |  | 0 | 0.0% |  |  |
|  | UFT only | 134 | 51.1% |  | 0 | 0.0% |  |  |
|  | Paclitaxel then UFT | 0 | 0.0% |  | 130 | 49.1% |  |  |
|  | Paclitaxel then S-1 | 0 | 0.0% |  | 135 | 50.9% |  |  |
| Age |  |  |  |  |  |  |  |  |
|  | <65y | 131 | 50.0% |  | 115 | 43.4% |  | 0.14 |
|  | ≥65y | 131 | 50.0% |  | 150 | 56.6% |  |  |
| Sex |  |  |  |  |  |  |  |  |
|  | Man | 174 | 66.4% |  | 187 | 70.6% |  | 0.04 |
|  | Woman | 88 | 33.6% |  | 78 | 29.4% |  |  |
| Body mass index (kg/m^2^) | | 20.6 | 3.0 |  | 20.8 | 2.9 |  | 0.54 |
| Histologic subtype | |  |  |  |  |  |  | 0.72 |
|  | Differentiated | 99 | 38.4% |  | 104 | 40.0% |  |  |
|  | Undifferentiated | 159 | 61.6% |  | 156 | 60.0% |  |  |
| T stage | |  |  |  |  |  |  | 0.26 |
|  | T1/T2 | 90 | 34.4% |  | 78 | 29.7% |  |  |
|  | T3/T4 | 172 | 65.6% |  | 185 | 70.3% |  |  |
| N stage | |  |  |  |  |  |  | 0.07 |
|  | N0 | 44 | 16.8% |  | 65 | 24.8% |  |  |
|  | N1 | 107 | 40.8% |  | 100 | 38.2% |  |  |
|  | N2/3 | 111 | 42.4% |  | 97 | 37.0% |  |  |
| TNM stage | |  |  |  |  |  |  | 0.68 |
|  | IA to II | 91 | 34.7% |  | 101 | 38.5% |  |  |
|  | IIIA | 74 | 28.2% |  | 70 | 26.7% |  |  |
|  | IIIB to IV | 97 | 37.0% |  | 91 | 34.7% |  |  |
| Low gene expression | |  |  |  |  |  |  |  |
|  | *VSNL1* | 185 | 70.6% |  | 190 | 71.7% |  | 0.85 |
|  | *CD44v* | 135 | 51.5% |  | 126 | 47.5% |  | 0.38 |
