# Supplementary material for: Gastric cancer biomarker analysis in patients treated with different adjuvant chemotherapy regimens within SAMIT, a phase III randomized controlled trial
Source: Sci Rep. 2022 May 20;12:8509. doi: 10.1038/s41598-022-12439-3 (PMC9123164; doi:10.1038/s41598-022-12439-3)
Supplement: Supplementary file 10 — Supplementary Table S2. [file 41598_2022_12439_MOESM10_ESM.docx]

**Supplementary Table S2.** Ranking of all genes according to interaction p-values for benefits from sequential paclitaxel in separate Cox or Fine-Gray regression models adjusted for clinical and pathological characteristics for overall survival, disease-free survival, and cumulative incidence of relapse

|  | **Overall survival** | |  | **Disease-free survival** | |  | **Cumulative incidence of relapse** | |
| --- | --- | --- | --- | --- | --- | --- | --- | --- |
| ***Gene*** | **Rank** | ***p*** |  | **Rank** | ***p*** |  | **Rank** | ***p*** |
| ***VSNL1*** | **1** | **0.00** |  | **1** | **0.01** |  | **2** | **0.03** |
| ***CD44v*** | **2** | **0.01** |  | **2** | **0.01** |  | **1** | **0.02** |
| *MTHFR* | 3 | 0.01 |  | 19 | 0.27 |  | 30 | 0.36 |
| *CDH17* | 4 | 0.03 |  | 8 | 0.08 |  | 6 | 0.06 |
| *AREG* | 5 | 0.03 |  | 7 | 0.08 |  | 7 | 0.07 |
| *MSI1* | 6 | 0.07 |  | 28 | 0.33 |  | 62 | 0.67 |
| *CXCR4* | 7 | 0.07 |  | 43 | 0.45 |  | 46 | 0.46 |
| *IGF2* | 8 | 0.08 |  | 4 | 0.04 |  | 13 | 0.14 |
| *CDKN2A* | 9 | 0.09 |  | 34 | 0.36 |  | 22 | 0.22 |
| *MMP14* | 10 | 0.13 |  | 36 | 0.38 |  | 34 | 0.38 |
| *MUC2* | 11 | 0.14 |  | 13 | 0.22 |  | 5 | 0.06 |
| *INHBA* | 12 | 0.17 |  | 6 | 0.06 |  | 9 | 0.11 |
| *ERBB3* | 13 | 0.19 |  | 84 | 0.80 |  | 86 | 0.87 |
| *REG4* | 14 | 0.19 |  | 32 | 0.36 |  | 92 | 0.91 |
| *UMPS* | 15 | 0.21 |  | 49 | 0.51 |  | 67 | 0.73 |
| *ZDHHC14* | 16 | 0.22 |  | 14 | 0.24 |  | 25 | 0.29 |
| *LGALS4* | 17 | 0.23 |  | 44 | 0.46 |  | 47 | 0.47 |
| *RRM2* | 18 | 0.24 |  | 65 | 0.66 |  | 32 | 0.37 |
| *ANGPT2* | 19 | 0.25 |  | 69 | 0.70 |  | 98 | 0.94 |
| *PDL1* | 20 | 0.26 |  | 42 | 0.45 |  | 15 | 0.18 |
| *TIMP_1* | 21 | 0.26 |  | 5 | 0.06 |  | 4 | 0.04 |
| *LGR5* | 22 | 0.26 |  | 18 | 0.26 |  | 23 | 0.24 |
| *DUT* | 23 | 0.26 |  | 60 | 0.63 |  | 50 | 0.49 |
| *CDX2* | 24 | 0.26 |  | 57 | 0.57 |  | 74 | 0.79 |
| *PLAU* | 25 | 0.27 |  | 9 | 0.13 |  | 12 | 0.14 |
| *GZMA* | 26 | 0.29 |  | 38 | 0.40 |  | 35 | 0.38 |
| *DHFR* | 27 | 0.30 |  | 50 | 0.51 |  | 43 | 0.45 |
| *TM9SF3* | 28 | 0.30 |  | 21 | 0.29 |  | 24 | 0.26 |
| *PDL2* | 29 | 0.30 |  | 26 | 0.30 |  | 10 | 0.13 |
| *PIK3CA* | 30 | 0.31 |  | 91 | 0.87 |  | 71 | 0.74 |
| *ITGB3* | 31 | 0.32 |  | 3 | 0.03 |  | 11 | 0.14 |
| *RRM1* | 32 | 0.32 |  | 15 | 0.24 |  | 58 | 0.66 |
| *MGMT* | 33 | 0.33 |  | 20 | 0.28 |  | 21 | 0.22 |
| *PLA2G2A* | 34 | 0.34 |  | 64 | 0.66 |  | 57 | 0.64 |
| *CAV1* | 35 | 0.35 |  | 59 | 0.59 |  | 91 | 0.90 |
| *DAPK1* | 36 | 0.35 |  | 55 | 0.55 |  | 99 | 0.95 |
| *SPARC* | 37 | 0.35 |  | 17 | 0.25 |  | 20 | 0.21 |
| *THBS1* | 38 | 0.36 |  | 71 | 0.71 |  | 83 | 0.85 |
| *GADD45A* | 39 | 0.36 |  | 52 | 0.54 |  | 38 | 0.42 |
| *MMP11* | 40 | 0.39 |  | 23 | 0.29 |  | 8 | 0.10 |
| *PTEN* | 41 | 0.39 |  | 104 | 0.98 |  | 77 | 0.82 |
| *CCR7* | 42 | 0.40 |  | 16 | 0.25 |  | 42 | 0.44 |
| *HPSE* | 43 | 0.42 |  | 35 | 0.37 |  | 44 | 0.46 |
| *RUNX3* | 44 | 0.46 |  | 62 | 0.65 |  | 26 | 0.31 |
| *CLDN4* | 45 | 0.47 |  | 72 | 0.72 |  | 100 | 0.97 |
| *MMP10* | 46 | 0.47 |  | 58 | 0.59 |  | 54 | 0.60 |
| *BCL2* | 47 | 0.47 |  | 51 | 0.52 |  | 80 | 0.83 |
| *FAS* | 48 | 0.48 |  | 45 | 0.47 |  | 88 | 0.88 |
| *OLFM4* | 49 | 0.49 |  | 30 | 0.35 |  | 19 | 0.20 |
| *EZH2* | 50 | 0.50 |  | 25 | 0.30 |  | 17 | 0.19 |
| *MMP9* | 51 | 0.50 |  | 39 | 0.42 |  | 37 | 0.39 |
| *ABCC1* | 52 | 0.50 |  | 24 | 0.29 |  | 55 | 0.61 |
| *FPGS* | 53 | 0.52 |  | 54 | 0.54 |  | 63 | 0.68 |
| *TGFA* | 54 | 0.53 |  | 103 | 0.98 |  | 102 | 0.98 |
| *DPD* | 55 | 0.54 |  | 75 | 0.74 |  | 70 | 0.74 |
| *ERCC1* | 56 | 0.56 |  | 76 | 0.74 |  | 52 | 0.54 |
| *KDR* | 57 | 0.59 |  | 63 | 0.66 |  | 79 | 0.82 |
| *TYMP* | 58 | 0.60 |  | 100 | 0.92 |  | 75 | 0.79 |
| *BCL2xL* | 59 | 0.60 |  | 73 | 0.72 |  | 68 | 0.73 |
| *HDAC1* | 60 | 0.61 |  | 61 | 0.64 |  | 40 | 0.42 |
| *ABCB1* | 61 | 0.62 |  | 96 | 0.89 |  | 87 | 0.88 |
| *MUC13* | 62 | 0.63 |  | 37 | 0.38 |  | 56 | 0.62 |
| *MAPT* | 63 | 0.64 |  | 80 | 0.78 |  | 97 | 0.93 |
| *TS* | 64 | 0.65 |  | 70 | 0.70 |  | 78 | 0.82 |
| *CCND1* | 65 | 0.66 |  | 31 | 0.35 |  | 45 | 0.46 |
| *ABCG2* | 66 | 0.66 |  | 95 | 0.89 |  | 60 | 0.66 |
| *SEMA3B* | 67 | 0.67 |  | 78 | 0.75 |  | 85 | 0.87 |
| *ESR1* | 68 | 0.68 |  | 102 | 0.97 |  | 105 | 0.99 |
| *MMP2* | 69 | 0.69 |  | 67 | 0.68 |  | 27 | 0.31 |
| *TSPAN8* | 70 | 0.70 |  | 33 | 0.36 |  | 84 | 0.85 |
| *CLDN3* | 71 | 0.70 |  | 11 | 0.21 |  | 49 | 0.47 |
| *CLDN18* | 72 | 0.71 |  | 89 | 0.85 |  | 65 | 0.71 |
| *EGF* | 73 | 0.73 |  | 101 | 0.93 |  | 101 | 0.98 |
| *EGFR* | 74 | 0.75 |  | 81 | 0.79 |  | 66 | 0.73 |
| *PDGFRB* | 75 | 0.76 |  | 47 | 0.49 |  | 41 | 0.43 |
| *TOP1* | 76 | 0.76 |  | 82 | 0.79 |  | 94 | 0.92 |
| *APOE* | 77 | 0.78 |  | 41 | 0.44 |  | 14 | 0.18 |
| *BIRC5* | 78 | 0.78 |  | 97 | 0.90 |  | 93 | 0.91 |
| *PROM1* | 79 | 0.79 |  | 12 | 0.22 |  | 16 | 0.19 |
| *TOP2A* | 80 | 0.81 |  | 93 | 0.88 |  | 96 | 0.93 |
| *FGFR2* | 81 | 0.81 |  | 22 | 0.29 |  | 89 | 0.89 |
| *ERBB2* | 82 | 0.82 |  | 105 | 0.99 |  | 53 | 0.59 |
| *PTGS2* | 83 | 0.82 |  | 48 | 0.49 |  | 29 | 0.32 |
| *MLH1* | 84 | 0.83 |  | 90 | 0.87 |  | 76 | 0.80 |
| *DSG2* | 85 | 0.86 |  | 87 | 0.83 |  | 82 | 0.84 |
| *SEC11A* | 86 | 0.86 |  | 85 | 0.80 |  | 90 | 0.90 |
| *IGF1R* | 87 | 0.88 |  | 56 | 0.56 |  | 39 | 0.42 |
| *APC* | 88 | 0.89 |  | 88 | 0.83 |  | 73 | 0.76 |
| *JAK2* | 89 | 0.90 |  | 53 | 0.54 |  | 51 | 0.51 |
| *MIA* | 90 | 0.90 |  | 68 | 0.69 |  | 95 | 0.92 |
| *GGH* | 91 | 0.91 |  | 27 | 0.31 |  | 28 | 0.32 |
| *PECAM1* | 92 | 0.92 |  | 77 | 0.74 |  | 61 | 0.67 |
| *VEGFA* | 93 | 0.93 |  | 98 | 0.91 |  | 69 | 0.74 |
| *MST1R* | 94 | 0.93 |  | 74 | 0.74 |  | 104 | 0.99 |
| *E2F1* | 95 | 0.94 |  | 99 | 0.92 |  | 81 | 0.83 |
| *VCAM1* | 96 | 0.95 |  | 46 | 0.48 |  | 48 | 0.47 |
| *NANOG* | 97 | 0.96 |  | 10 | 0.15 |  | 18 | 0.20 |
| *CLDN7* | 98 | 0.97 |  | 94 | 0.88 |  | 36 | 0.39 |
| *SIRT1* | 99 | 0.97 |  | 83 | 0.79 |  | 31 | 0.37 |
| *EREG* | 100 | 0.98 |  | 66 | 0.68 |  | 59 | 0.66 |
| *LDHA* | 101 | 0.98 |  | 40 | 0.43 |  | 103 | 0.98 |
| *BAX* | 102 | 0.99 |  | 86 | 0.81 |  | 33 | 0.38 |
| *UPP1* | 103 | 0.99 |  | 29 | 0.34 |  | 3 | 0.03 |
| *MMP7* | 104 | 0.99 |  | 92 | 0.88 |  | 72 | 0.76 |
| *HGF* | 105 | 1.00 |  | 79 | 0.77 |  | 64 | 0.69 |
