# Supplementary material for: Gastric cancer biomarker analysis in patients treated with different adjuvant chemotherapy regimens within SAMIT, a phase III randomized controlled trial
Source: Sci Rep. 2022 May 20;12:8509. doi: 10.1038/s41598-022-12439-3 (PMC9123164; doi:10.1038/s41598-022-12439-3)
Supplement: Supplementary file 11 — Supplementary Table S3. [file 41598_2022_12439_MOESM11_ESM.docx]

**Supplementary Table S3.** Added predictive performance in the Cox regression model considering the interaction between predictive biomarkers and the treatment group, and clinical and pathological characteristics for overall survival

| **Gene** | **Harrell's C 0.632+ estimator** |  | **Harrell's C apparent estimator** |
| --- | --- | --- | --- |
| *VSNL1* | 0.7111 |  | 0.7266 |
| *CD44v* | 0.7083 |  | 0.7252 |
| *MTHFR* | 0.7123 |  | 0.7261 |
| *CDH17* | 0.7105 |  | 0.7242 |
| *AREG* | 0.7108 |  | 0.7232 |
| *MSI1* | 0.7085 |  | 0.7233 |
| *CXCR4* | 0.7093 |  | 0.7233 |
| *IGF2* | 0.7086 |  | 0.7236 |
| *CDKN2A* | 0.7079 |  | 0.7219 |
| *MMP14* | 0.7064 |  | 0.7203 |
| *MUC2* | 0.7085 |  | 0.7210 |
| *INHBA* | 0.7082 |  | 0.7222 |
| *ERBB3* | 0.7063 |  | 0.7199 |
| *REG4* | 0.7071 |  | 0.7229 |
| *UMPS* | 0.7082 |  | 0.7235 |
| *ZDHHC14* | 0.7054 |  | 0.7209 |
| *LGALS4* | 0.7069 |  | 0.7193 |
| *RRM2* | 0.7057 |  | 0.7211 |
| *ANGPT2* | 0.7055 |  | 0.7201 |
| *PDL1* | 0.7060 |  | 0.7200 |
| *TIMP_1* | 0.7048 |  | 0.7205 |
| *LGR5* | 0.7056 |  | 0.7205 |
| *DUT* | 0.7077 |  | 0.7215 |
| *CDX2* | 0.7073 |  | 0.7215 |
| *PLAU* | 0.7061 |  | 0.7219 |
| *GZMA* | 0.7123 |  | 0.7251 |
| *DHFR* | 0.7061 |  | 0.7204 |
| *TM9SF3* | 0.7046 |  | 0.7186 |
| *PDL2* | 0.7074 |  | 0.7220 |
| *PIK3CA* | 0.7069 |  | 0.7194 |
| *ITGB3* | 0.7069 |  | 0.7207 |
| *RRM1* | 0.7169 |  | 0.7338 |
| *MGMT* | 0.7067 |  | 0.7224 |
| *PLA2G2A* | 0.7054 |  | 0.7187 |
| *CAV1* | 0.7072 |  | 0.7215 |
| *DAPK1* | 0.7049 |  | 0.7190 |
| *SPARC* | 0.7055 |  | 0.7203 |
| *THBS1* | 0.7046 |  | 0.7201 |
| *GADD45A* | 0.7038 |  | 0.7185 |
| *MMP11* | 0.7050 |  | 0.7183 |
| *PTEN* | 0.7055 |  | 0.7217 |
| *CCR7* | 0.7077 |  | 0.7224 |
| *HPSE* | 0.7053 |  | 0.7199 |
| *RUNX3* | 0.7044 |  | 0.7189 |
| *CLDN4* | 0.7096 |  | 0.7232 |
| *MMP10* | 0.7098 |  | 0.7262 |
| *BCL2* | 0.7063 |  | 0.7195 |
| *FAS* | 0.7050 |  | 0.7201 |
| *OLFM4* | 0.7064 |  | 0.7201 |
| *EZH2* | 0.7035 |  | 0.7182 |
| *MMP9* | 0.7049 |  | 0.7203 |
| *ABCC1* | 0.7085 |  | 0.7218 |
| *FPGS* | 0.7049 |  | 0.7179 |
| *TGFA* | 0.7045 |  | 0.7189 |
| *DPD* | 0.7062 |  | 0.7188 |
| *ERCC1* | 0.7052 |  | 0.7195 |
| *KDR* | 0.7053 |  | 0.7192 |
| *TYMP* | 0.7046 |  | 0.7189 |
| *BCL2L11* | 0.7109 |  | 0.7257 |
| *HDAC1* | 0.7040 |  | 0.7184 |
| *ABCB1* | 0.7025 |  | 0.7181 |
| *MUC13* | 0.7057 |  | 0.7201 |
| *MAPT* | 0.7046 |  | 0.7198 |
| *TS* | 0.7037 |  | 0.7189 |
| *CCND1* | 0.7048 |  | 0.7206 |
| *ABCG2* | 0.7033 |  | 0.7188 |
| *SEMA3B* | 0.7035 |  | 0.7203 |
| *ESR1* | 0.7052 |  | 0.7189 |
| *MMP2* | 0.7048 |  | 0.7188 |
| *TSPAN8* | 0.7047 |  | 0.7205 |
| *CLDN3* | 0.7038 |  | 0.7183 |
| *CLDN18* | 0.7047 |  | 0.7200 |
| *EGF* | 0.7048 |  | 0.7189 |
| *EGFR* | 0.7040 |  | 0.7183 |
| *PDGFRB* | 0.7039 |  | 0.7189 |
| *TOP1* | 0.7049 |  | 0.7227 |
| *APOE* | 0.7021 |  | 0.7180 |
| *BIRC5* | 0.7067 |  | 0.7217 |
| *PROM1* | 0.7084 |  | 0.7213 |
| *TOP2A* | 0.7031 |  | 0.7197 |
| *FGFR2* | 0.7034 |  | 0.7188 |
| *ERBB2* | 0.7082 |  | 0.7224 |
| *PTGS2* | 0.7092 |  | 0.7224 |
| *MLH1* | 0.7032 |  | 0.7167 |
| *DSG2* | 0.7094 |  | 0.7242 |
| *SEC11A* | 0.7036 |  | 0.7183 |
| *IGF1R* | 0.7127 |  | 0.7273 |
| *APC* | 0.7052 |  | 0.7192 |
| *JAK2* | 0.7051 |  | 0.7188 |
| *MIA* | 0.7037 |  | 0.7186 |
| *GGH* | 0.7029 |  | 0.7179 |
| *PECAM1* | 0.7054 |  | 0.7202 |
| *VEGFA* | 0.7026 |  | 0.7183 |
| *MST1R* | 0.7040 |  | 0.7188 |
| *E2F1* | 0.7031 |  | 0.7189 |
| *VCAM1* | 0.7030 |  | 0.7182 |
| *NANOG* | 0.7021 |  | 0.7189 |
| *CLDN7* | 0.7041 |  | 0.7191 |
| *SIRT1* | 0.7043 |  | 0.7190 |
| *EREG* | 0.7039 |  | 0.7206 |
| *LDHA* | 0.7055 |  | 0.7212 |
| *BAX* | 0.7011 |  | 0.7184 |
| *UPP1* | 0.7018 |  | 0.7176 |
| *MMP7* | 0.7012 |  | 0.7186 |
| *HGF* | 0.7006 |  | 0.7185 |
