# Supplementary material for: Gastric cancer biomarker analysis in patients treated with different adjuvant chemotherapy regimens within SAMIT, a phase III randomized controlled trial
Source: Sci Rep. 2022 May 20;12:8509. doi: 10.1038/s41598-022-12439-3 (PMC9123164; doi:10.1038/s41598-022-12439-3)
Supplement: Supplementary file 12 — Supplementary Table S4. [file 41598_2022_12439_MOESM12_ESM.docx]

**Supplementary Table** **S4.** Comparison between VSNL1 or CD44 mRNA expression and clinicopathological factors.

|  |  | **VSNL1 mRNA expression(*n*=527)** | |  | **CD44v mRNA expression(n=527)** | |  |
| --- | --- | --- | --- | --- | --- | --- | --- |
|  |  | **Low (n=375)** | **High (n=152)** | ***p*-value** | **Low (n=261)** | **High (n=266)** | ***p*-value** |
| Arms |  |  |  |  |  |  |  |
|  | S-1 or UFT only | 186 | 76 |  | 133 | 129 |  |
|  | Sequential paclitaxel | 189 | 76 |  | 128 | 137 |  |
| 5-Year Survival | |  |  |  |  |  |  |
|  | S-1 or UFT only | 26.4 | 24.6 |  | 23.0 | 29.1 |  |
|  | Sequential paclitaxel | 34.2 | 21.3 |  | 38.1 | 23.7 |  |
| Age |  |  |  |  |  |  |  |
|  | <65 years | 188 | 55 | **0.0036** | 144 | 134 | 0.2701 |
|  | ≥65 years | 187 | 97 |  | 117 | 132 |  |
| Sex |  |  |  |  |  |  |  |
|  | male | 254 | 106 | 0.6543 | 175 | 185 | 0.5376 |
|  | female | 121 | 46 |  | 86 | 81 |  |
| PS | |  |  |  |  |  |  |
|  | 0 | 328 | 114 | 0.6610 | 224 | 218 | 0.2273 |
|  | 1 | 47 | 38 |  | 37 | 48 |  |
|  | 2 or 3 | 0 | 0 |  | 0 | 0 |  |
| Tumor Location | |  |  |  |  |  |  |
|  | T | 9 | 3 | 0.5888 | 5 | 7 | 0.8060 |
|  | U | 99 | 32 |  | 68 | 63 |  |
|  | M | 121 | 55 |  | 89 | 87 |  |
|  | L | 146 | 62 |  | 99 | 109 |  |
| Tumor diameter | |  |  |  |  |  |  |
|  | <65 | 199 | 79 | 0.8199 | 144 | 134 | 0.2701 |
|  | ≧65 | 176 | 73 |  | 117 | 132 |  |
| Histopathological type | |  |  |  |  |  |  |
|  | Differentiated adenocarcinoma | 140 | 72 | **0.0026** | 112 | 100 | 0.1555 |
|  | Undifferentiated adenocarcinoma | 234 | 76 |  | 145 | 165 |  |
|  | Others | 1 | 4 |  | 4 | 1 |  |
| pT | |  |  |  |  |  |  |
|  | 1 | 6 | 1 | 0.3798 | 5 | 2 | 0.2819 |
|  | 2 | 107 | 54 |  | 75 | 86 |  |
|  | 3 | 247 | 92 |  | 168 | 171 |  |
|  | 4 | 15 | 5 |  | 13 | 7 |  |
| pN | |  |  |  |  |  |  |
|  | 0 | 79 | 30 | 0.1190 | 62 | 47 | 0.2113 |
|  | 1 | 58 | 32 |  | 44 | 44 |  |
|  | 2 | 79 | 40 |  | 60 | 59 |  |
|  | 3 | 159 | 50 |  | 93 | 116 |  |
| pTNM stage | |  |  |  |  |  |  |
|  | I | 29 (7.7) | 8 (5.3) | **0.0180** | 18 (5.0) | 19 (7.1) | 0.1272 |
|  | IIA | 68 (18.1) | 39 (25.7) |  | 64 (24.5) | 43 (16.2) |  |
|  | IIB | 73 (19.4) | 33 (21.7) |  | 47 (18.0) | 59 (22.2) |  |
|  | IIIA | 72 (19.2) | 33 (21.7) |  | 55 (21.1) | 50 (18.8) |  |
|  | IIIB | 71 (18.9) | 30 (19.7) |  | 42 (16.1) | 59 (22.2) |  |
|  | IIIC | 62 (16.5) | 9 (5.9) |  | 36 (13.8) | 36 (13.5) |  |

VSNL1, Visinin-like protein 1; CD44v, [CD44 variant isoforms; T, total; U, upper third, M, middle third, L, lower third; pT, pathological tumor depth; pN, pathological lymph node metastasis](https://www.ncbi.nlm.nih.gov/pmc/articles/PMC2119889/)
