# Supplementary material for: Gastric cancer biomarker analysis in patients treated with different adjuvant chemotherapy regimens within SAMIT, a phase III randomized controlled trial
Source: Sci Rep. 2022 May 20;12:8509. doi: 10.1038/s41598-022-12439-3 (PMC9123164; doi:10.1038/s41598-022-12439-3)
Supplement: Supplementary file 13 — Supplementary Table S5. [file 41598_2022_12439_MOESM13_ESM.docx]

**Supplementary Table S5.** Univariate and multivariate analyses of overall survival in patients with gastric cancer (n=527).

|  |  |  | Univariate |  |  |  | Multivariate |  |
| --- | --- | --- | --- | --- | --- | --- | --- | --- |
| Variables/categories | N | HR | 95% CI | *p*-Value |  | HR | 95% CI | *p*-Value |
| Age (years) |  |  |  | 0.741 |  |  |  |  |
| <65 | 266 | 1 |  |  |  |  |  |  |
| ≥65 | 147 | 0.954 | 0.723-1.260 |  |  |  |  |  |
| Sex |  |  |  | **0.048** |  |  |  | **0.0317** |
| Male | 293 | 1 |  |  |  | 1 |  |  |
| Female | 120 | 1.370 | 1.003–1.871 |  |  | 1.417 | 1.031-1.947 |  |
| PS |  |  |  | **0.040** |  |  |  | 0.1783 |
| 0 | 442 | 1 |  |  |  | 1 |  |  |
| 1 | 85 | 0.693 | 0.488-0.953 |  |  | 0.784 | 0.551-1.117 |  |
| Tumour diameter |  |  |  | **<0.0001** |  |  |  | 0.0699 |
| <65 mm | 278 | 1 |  |  |  | 1 |  |  |
| ≥65 mm | 249 | 0.521 | 0.393-0.691 |  |  | 0.759 | 0.564-1.023 |  |
| Lauren’s classification |  |  |  | **0.037** |  |  |  | 0.1537 |
| Intestinal type | 212 | 1 |  |  |  | 1 |  |  |
| Diffuse type | 315 | 1.362 | 1.019-1.821 |  |  | 1.242 | 0.922-1.671 |  |
| pT |  |  |  | **<0.0001** |  |  |  | **<0.0001** |
| T1, T2, | 168 | 1 |  |  |  | 1 |  |  |
| T3, T4 | 359 | 0.394 | 0.260-0.539 |  |  | 0.428 | 0.295-0.621 |  |
| pN |  |  |  | **<0.0001** |  |  |  | **<0.0001** |
| N0, N1 | 199 | 1 |  |  |  | 1 |  |  |
| N2, N3 | 328 | 0.291 | 0.203-0.415 |  |  | 0.336 | 0.233-0.485 |  |
| Lymph node dissection |  |  |  | 0.873 |  |  |  |  |
| D1, D1+ | 23 | 1 |  |  |  |  |  |  |
| D2, D3 | 504 | 1.056 | 0.541-2.061 |  |  |  |  |  |
| VSNL1 mRNA |  |  |  | 0.255 |  |  |  |  |
| High expression | 152 | 1 |  |  |  |  |  |  |
| Low expression | 375 | 1.203 | 0.875-1.653 |  |  |  |  |  |
| CD44 mRNA |  |  |  | 0.430 |  |  |  |  |
| High expression | 266 | 1 |  |  |  |  |  |  |
| Low expression | 261 | 1.118 | 0.848-1.473 |  |  |  |  |  |

N, Number of patients; HR, hazard ratio; CI, confidence interval; VSNL1, visinin-like protein 1
