# Supplementary material for: Gastric cancer biomarker analysis in patients treated with different adjuvant chemotherapy regimens within SAMIT, a phase III randomized controlled trial
Source: Sci Rep. 2022 May 20;12:8509. doi: 10.1038/s41598-022-12439-3 (PMC9123164; doi:10.1038/s41598-022-12439-3)
Supplement: Supplementary file 14 — Supplementary Table S6. [file 41598_2022_12439_MOESM14_ESM.docx]

**Supplementary Table S6.** The 105 genes that were investigated, and the functional annotation of each gene using DAVID 6.7

| ID | Gene Name | Species | KEGG_PATHWAY |
| --- | --- | --- | --- |
| *APC* | APC, WNT signaling pathway regulator(APC) | *Homo sapiens* | hsa04310:Wnt signaling pathway,hsa04390:Hippo signaling pathway,hsa04550:Signaling pathways regulating pluripotency of stem cells,hsa04810:Regulation of actin cytoskeleton,hsa05166:HTLV-I infection,hsa05200:Pathways in cancer,hsa05206:MicroRNAs in cancer,hsa05210:Colorectal cancer,hsa05213:Endometrial cancer,hsa05217:Basal cell carcinoma, |
| *ABCB1* | ATP binding cassette subfamily B member 1(ABCB1) | *Homo sapiens* | hsa02010:ABC transporters,hsa04976:Bile secretion,hsa05206:MicroRNAs in cancer, |
| *ABCC1* | ATP binding cassette subfamily C member 1(ABCC1) | *Homo sapiens* | hsa02010:ABC transporters,hsa04071:Sphingolipid signaling pathway,hsa04977:Vitamin digestion and absorption,hsa05206:MicroRNAs in cancer, |
| *ABCG2* | ATP binding cassette subfamily G member 2 (Junior blood group)(ABCG2) | *Homo sapiens* | hsa02010:ABC transporters,hsa04976:Bile secretion, |
| *BAX* | BCL2 associated X, apoptosis regulator(BAX) | *Homo sapiens* | hsa04071:Sphingolipid signaling pathway,hsa04115:p53 signaling pathway,hsa04141:Protein processing in endoplasmic reticulum,hsa04210:Apoptosis,hsa04722:Neurotrophin signaling pathway,hsa04932:Non-alcoholic fatty liver disease (NAFLD),hsa05014:Amyotrophic lateral sclerosis (ALS),hsa05016:Huntington's disease,hsa05020:Prion diseases,hsa05152:Tuberculosis,hsa05161:Hepatitis B,hsa05166:HTLV-I infection,hsa05200:Pathways in cancer,hsa05203:Viral carcinogenesis,hsa05210:Colorectal cancer, |
| *BCLxL* | BCLxL, apoptosis regulator (BCLxL) | *Homo sapiens* | hsa04068:FoxO signaling pathway,hsa04151:PI3K-Akt signaling pathway,hsa04932:Non-alcoholic fatty liver disease (NAFLD),hsa05206:MicroRNAs in cancer, |
| *BCL2* | BCL2, apoptosis regulator(BCL2) | *Homo sapiens* | hsa04064:NF-kappa B signaling pathway,hsa04066:HIF-1 signaling pathway,hsa04071:Sphingolipid signaling pathway,hsa04141:Protein processing in endoplasmic reticulum,hsa04151:PI3K-Akt signaling pathway,hsa04210:Apoptosis,hsa04261:Adrenergic signaling in cardiomyocytes,hsa04510:Focal adhesion,hsa04722:Neurotrophin signaling pathway,hsa04725:Cholinergic synapse,hsa05014:Amyotrophic lateral sclerosis (ALS),hsa05145:Toxoplasmosis,hsa05152:Tuberculosis,hsa05161:Hepatitis B,hsa05169:Epstein-Barr virus infection,hsa05200:Pathways in cancer,hsa05206:MicroRNAs in cancer,hsa05210:Colorectal cancer,hsa05215:Prostate cancer,hsa05222:Small cell lung cancer, |
| *CCR7* | C-C motif chemokine receptor 7(CCR7) | *Homo sapiens* | hsa04060:Cytokine-cytokine receptor interaction,hsa04062:Chemokine signaling pathway, |
| *CXCR4* | C-X-C motif chemokine receptor 4(CXCR4) | *Homo sapiens* | hsa04060:Cytokine-cytokine receptor interaction,hsa04062:Chemokine signaling pathway,hsa04144:Endocytosis,hsa04360:Axon guidance,hsa04670:Leukocyte transendothelial migration,hsa04672:Intestinal immune network for IgA production,hsa05200:Pathways in cancer, |
| *CD44v* | CD44 molecule (Indian blood group)(CD44) | *Homo sapiens* | hsa04512:ECM-receptor interaction,hsa04640:Hematopoietic cell lineage,hsa05131:Shigellosis,hsa05169:Epstein-Barr virus infection,hsa05205:Proteoglycans in cancer,hsa05206:MicroRNAs in cancer, |
| *E2F1* | E2F transcription factor 1(E2F1) | *Homo sapiens* | hsa04110:Cell cycle,hsa05161:Hepatitis B,hsa05166:HTLV-I infection,hsa05200:Pathways in cancer,hsa05206:MicroRNAs in cancer,hsa05212:Pancreatic cancer,hsa05214:Glioma,hsa05215:Prostate cancer,hsa05218:Melanoma,hsa05219:Bladder cancer,hsa05220:Chronic myeloid leukemia,hsa05222:Small cell lung cancer,hsa05223:Non-small cell lung cancer, |
| *ERCC1* | ERCC excision repair 1, endonuclease non-catalytic subunit(ERCC1) | *Homo sapiens* | hsa03420:Nucleotide excision repair,hsa03460:Fanconi anemia pathway, |
| *FAS* | Fas cell surface death receptor(FAS) | *Homo sapiens* | hsa04010:MAPK signaling pathway,hsa04060:Cytokine-cytokine receptor interaction,hsa04115:p53 signaling pathway,hsa04210:Apoptosis,hsa04650:Natural killer cell mediated cytotoxicity,hsa04668:TNF signaling pathway,hsa04932:Non-alcoholic fatty liver disease (NAFLD),hsa04940:Type I diabetes mellitus,hsa05010:Alzheimer's disease,hsa05142:Chagas disease (American trypanosomiasis),hsa05143:African trypanosomiasis,hsa05161:Hepatitis B,hsa05162:Measles,hsa05164:Influenza A,hsa05168:Herpes simplex infection,hsa05200:Pathways in cancer,hsa05205:Proteoglycans in cancer,hsa05320:Autoimmune thyroid disease,hsa05330:Allograft rejection,hsa05332:Graft-versus-host disease, |
| *JAK2* | Janus kinase 2(JAK2) | *Homo sapiens* | hsa04062:Chemokine signaling pathway,hsa04151:PI3K-Akt signaling pathway,hsa04550:Signaling pathways regulating pluripotency of stem cells,hsa04630:Jak-STAT signaling pathway,hsa04725:Cholinergic synapse,hsa04917:Prolactin signaling pathway,hsa04920:Adipocytokine signaling pathway,hsa05140:Leishmaniasis,hsa05145:Toxoplasmosis,hsa05152:Tuberculosis,hsa05162:Measles,hsa05164:Influenza A,hsa05168:Herpes simplex infection, |
| *MET* | MET proto-oncogene, receptor tyrosine kinase(MET) | *Homo sapiens* | hsa04014:Ras signaling pathway,hsa04015:Rap1 signaling pathway,hsa04151:PI3K-Akt signaling pathway,hsa04360:Axon guidance,hsa04510:Focal adhesion,hsa04520:Adherens junction,hsa05100:Bacterial invasion of epithelial cells,hsa05120:Epithelial cell signaling in Helicobacter pylori infection,hsa05144:Malaria,hsa05200:Pathways in cancer,hsa05202:Transcriptional misregulation in cancer,hsa05205:Proteoglycans in cancer,hsa05206:MicroRNAs in cancer,hsa05211:Renal cell carcinoma,hsa05218:Melanoma,hsa05230:Central carbon metabolism in cancer, |
| *NANOG* | Nanog homeobox(NANOG) | *Homo sapiens* | hsa04550:Signaling pathways regulating pluripotency of stem cells,hsa05205:Proteoglycans in cancer, |
| *SEC11A* | SEC11 homolog A, signal peptidase complex subunit(SEC11A) | *Homo sapiens* | hsa03060:Protein export, |
| *TIMP1* | TIMP metallopeptidase inhibitor 1(TIMP1) | *Homo sapiens* | hsa04066:HIF-1 signaling pathway, |
| *AREG* | amphiregulin(AREG) | *Homo sapiens* | hsa04012:ErbB signaling pathway,hsa04390:Hippo signaling pathway, |
| *ANGPT2* | angiopoietin 2(ANGPT2) | *Homo sapiens* | hsa04014:Ras signaling pathway,hsa04015:Rap1 signaling pathway,hsa04066:HIF-1 signaling pathway,hsa04151:PI3K-Akt signaling pathway, |
| *APOE* | apolipoprotein E(APOE) | *Homo sapiens* | hsa05010:Alzheimer's disease, |
| *BIRC5* | baculoviral IAP repeat containing 5(BIRC5) | *Homo sapiens* | hsa04390:Hippo signaling pathway,hsa05161:Hepatitis B,hsa05200:Pathways in cancer,hsa05210:Colorectal cancer, |
| *CAV1* | caveolin 1(CAV1) | *Homo sapiens* | hsa04144:Endocytosis,hsa04510:Focal adhesion,hsa05100:Bacterial invasion of epithelial cells,hsa05205:Proteoglycans in cancer,hsa05416:Viral myocarditis, |
| *CLDN18* | claudin 18(CLDN18) | *Homo sapiens* | hsa04514:Cell adhesion molecules (CAMs),hsa04530:Tight junction,hsa04670:Leukocyte transendothelial migration,hsa05160:Hepatitis C, |
| *CLDN3* | claudin 3(CLDN3) | *Homo sapiens* | hsa04514:Cell adhesion molecules (CAMs),hsa04530:Tight junction,hsa04670:Leukocyte transendothelial migration,hsa05160:Hepatitis C, |
| *CLDN4* | claudin 4(CLDN4) | *Homo sapiens* | hsa04514:Cell adhesion molecules (CAMs),hsa04530:Tight junction,hsa04670:Leukocyte transendothelial migration,hsa05160:Hepatitis C, |
| *CLDN7* | claudin 7(CLDN7) | *Homo sapiens* | hsa04514:Cell adhesion molecules (CAMs),hsa04530:Tight junction,hsa04670:Leukocyte transendothelial migration,hsa05160:Hepatitis C, |
| *CCND1* | cyclin D1(CCND1) | *Homo sapiens* | hsa04068:FoxO signaling pathway,hsa04110:Cell cycle,hsa04115:p53 signaling pathway,hsa04151:PI3K-Akt signaling pathway,hsa04152:AMPK signaling pathway,hsa04310:Wnt signaling pathway,hsa04390:Hippo signaling pathway,hsa04510:Focal adhesion,hsa04630:Jak-STAT signaling pathway,hsa04917:Prolactin signaling pathway,hsa04919:Thyroid hormone signaling pathway,hsa04921:Oxytocin signaling pathway,hsa05161:Hepatitis B,hsa05162:Measles,hsa05166:HTLV-I infection,hsa05200:Pathways in cancer,hsa05203:Viral carcinogenesis,hsa05205:Proteoglycans in cancer,hsa05206:MicroRNAs in cancer,hsa05210:Colorectal cancer,hsa05212:Pancreatic cancer,hsa05213:Endometrial cancer,hsa05214:Glioma,hsa05215:Prostate cancer,hsa05216:Thyroid cancer,hsa05218:Melanoma,hsa05219:Bladder cancer,hsa05220:Chronic myeloid leukemia,hsa05221:Acute myeloid leukemia,hsa05222:Small cell lung cancer,hsa05223:Non-small cell lung cancer,hsa05416:Viral myocarditis, |
| *CDKN2A* | cyclin dependent kinase inhibitor 2A(CDKN2A) | *Homo sapiens* | hsa04110:Cell cycle,hsa04115:p53 signaling pathway,hsa05166:HTLV-I infection,hsa05200:Pathways in cancer,hsa05203:Viral carcinogenesis,hsa05206:MicroRNAs in cancer,hsa05212:Pancreatic cancer,hsa05214:Glioma,hsa05218:Melanoma,hsa05219:Bladder cancer,hsa05220:Chronic myeloid leukemia,hsa05223:Non-small cell lung cancer, |
| *DAPK1* | death associated protein kinase 1(DAPK1) | *Homo sapiens* | hsa05200:Pathways in cancer,hsa05219:Bladder cancer, |
| *DUT* | deoxyuridine triphosphatase(DUT) | *Homo sapiens* | hsa00240:Pyrimidine metabolism,hsa01100:Metabolic pathways, |
| *DSG2* | desmoglein 2(DSG2) | *Homo sapiens* | hsa05412:Arrhythmogenic right ventricular cardiomyopathy (ARVC), |
| *DHFR* | dihydrofolate reductase(DHFR) | *Homo sapiens* | hsa00670:One carbon pool by folate,hsa00790:Folate biosynthesis,hsa01100:Metabolic pathways, |
| *EZH2* | enhancer of zeste 2 polycomb repressive complex 2 subunit(EZH2) | *Homo sapiens* | hsa05206:MicroRNAs in cancer, |
| *EGFR* | epidermal growth factor receptor(EGFR) | *Homo sapiens* | hsa04010:MAPK signaling pathway,hsa04012:ErbB signaling pathway,hsa04014:Ras signaling pathway,hsa04015:Rap1 signaling pathway,hsa04020:Calcium signaling pathway,hsa04066:HIF-1 signaling pathway,hsa04068:FoxO signaling pathway,hsa04144:Endocytosis,hsa04151:PI3K-Akt signaling pathway,hsa04320:Dorso-ventral axis formation,hsa04510:Focal adhesion,hsa04520:Adherens junction,hsa04540:Gap junction,hsa04810:Regulation of actin cytoskeleton,hsa04912:GnRH signaling pathway,hsa04915:Estrogen signaling pathway,hsa04921:Oxytocin signaling pathway,hsa05120:Epithelial cell signaling in Helicobacter pylori infection,hsa05160:Hepatitis C,hsa05200:Pathways in cancer,hsa05205:Proteoglycans in cancer,hsa05206:MicroRNAs in cancer,hsa05212:Pancreatic cancer,hsa05213:Endometrial cancer,hsa05214:Glioma,hsa05215:Prostate cancer,hsa05218:Melanoma,hsa05219:Bladder cancer,hsa05223:Non-small cell lung cancer,hsa05230:Central carbon metabolism in cancer,hsa05231:Choline metabolism in cancer, |
| *EGF* | epidermal growth factor(EGF) | *Homo sapiens* | hsa04010:MAPK signaling pathway,hsa04012:ErbB signaling pathway,hsa04014:Ras signaling pathway,hsa04015:Rap1 signaling pathway,hsa04066:HIF-1 signaling pathway,hsa04068:FoxO signaling pathway,hsa04151:PI3K-Akt signaling pathway,hsa04510:Focal adhesion,hsa04540:Gap junction,hsa04810:Regulation of actin cytoskeleton,hsa05160:Hepatitis C,hsa05200:Pathways in cancer,hsa05212:Pancreatic cancer,hsa05213:Endometrial cancer,hsa05214:Glioma,hsa05215:Prostate cancer,hsa05218:Melanoma,hsa05219:Bladder cancer,hsa05223:Non-small cell lung cancer,hsa05231:Choline metabolism in cancer, |
| *EREG* | epiregulin(EREG) | *Homo sapiens* | hsa04012:ErbB signaling pathway, |
| *ERBB2* | erb-b2 receptor tyrosine kinase 2(ERBB2) | *Homo sapiens* | hsa04012:ErbB signaling pathway,hsa04020:Calcium signaling pathway,hsa04066:HIF-1 signaling pathway,hsa04510:Focal adhesion,hsa04520:Adherens junction,hsa05200:Pathways in cancer,hsa05205:Proteoglycans in cancer,hsa05206:MicroRNAs in cancer,hsa05212:Pancreatic cancer,hsa05213:Endometrial cancer,hsa05215:Prostate cancer,hsa05219:Bladder cancer,hsa05223:Non-small cell lung cancer,hsa05230:Central carbon metabolism in cancer, |
| *ERBB3* | erb-b2 receptor tyrosine kinase 3(ERBB3) | *Homo sapiens* | hsa04012:ErbB signaling pathway,hsa04020:Calcium signaling pathway,hsa05205:Proteoglycans in cancer,hsa05206:MicroRNAs in cancer, |
| *ESR1* | estrogen receptor 1(ESR1) | *Homo sapiens* | hsa04915:Estrogen signaling pathway,hsa04917:Prolactin signaling pathway,hsa04919:Thyroid hormone signaling pathway,hsa04961:Endocrine and other factor-regulated calcium reabsorption,hsa05205:Proteoglycans in cancer, |
| *FGFR2* | fibroblast growth factor receptor 2(FGFR2) | *Homo sapiens* | hsa04010:MAPK signaling pathway,hsa04014:Ras signaling pathway,hsa04015:Rap1 signaling pathway,hsa04144:Endocytosis,hsa04151:PI3K-Akt signaling pathway,hsa04550:Signaling pathways regulating pluripotency of stem cells,hsa04810:Regulation of actin cytoskeleton,hsa05200:Pathways in cancer,hsa05215:Prostate cancer,hsa05230:Central carbon metabolism in cancer, |
| *FPGS* | folylpolyglutamate synthase(FPGS) | *Homo sapiens* | hsa00790:Folate biosynthesis,hsa01100:Metabolic pathways, |
| *GGH* | gamma-glutamyl hydrolase(GGH) | *Homo sapiens* | hsa00790:Folate biosynthesis, |
| *GZMA* | granzyme A(GZMA) | *Homo sapiens* | hsa04080:Neuroactive ligand-receptor interaction, |
| *HPSE* | heparanase(HPSE) | *Homo sapiens* | hsa00531:Glycosaminoglycan degradation,hsa01100:Metabolic pathways,hsa05205:Proteoglycans in cancer, |
| *HGF* | hepatocyte growth factor(HGF) | *Homo sapiens* | hsa04014:Ras signaling pathway,hsa04015:Rap1 signaling pathway,hsa04151:PI3K-Akt signaling pathway,hsa04510:Focal adhesion,hsa05144:Malaria,hsa05200:Pathways in cancer,hsa05205:Proteoglycans in cancer,hsa05211:Renal cell carcinoma,hsa05218:Melanoma, |
| *HDAC1* | histone deacetylase 1(HDAC1) | *Homo sapiens* | hsa04110:Cell cycle,hsa04330:Notch signaling pathway,hsa04919:Thyroid hormone signaling pathway,hsa05016:Huntington's disease,hsa05034:Alcoholism,hsa05169:Epstein-Barr virus infection,hsa05200:Pathways in cancer,hsa05202:Transcriptional misregulation in cancer,hsa05203:Viral carcinogenesis,hsa05220:Chronic myeloid leukemia, |
| *INHBA* | inhibin beta A subunit(INHBA) | *Homo sapiens* | hsa04060:Cytokine-cytokine receptor interaction,hsa04350:TGF-beta signaling pathway,hsa04550:Signaling pathways regulating pluripotency of stem cells, |
| *IGF1R* | insulin like growth factor 1 receptor(IGF1R) | *Homo sapiens* | hsa04014:Ras signaling pathway,hsa04015:Rap1 signaling pathway,hsa04066:HIF-1 signaling pathway,hsa04068:FoxO signaling pathway,hsa04114:Oocyte meiosis,hsa04144:Endocytosis,hsa04151:PI3K-Akt signaling pathway,hsa04152:AMPK signaling pathway,hsa04510:Focal adhesion,hsa04520:Adherens junction,hsa04550:Signaling pathways regulating pluripotency of stem cells,hsa04730:Long-term depression,hsa04913:Ovarian steroidogenesis,hsa04914:Progesterone-mediated oocyte maturation,hsa05200:Pathways in cancer,hsa05202:Transcriptional misregulation in cancer,hsa05205:Proteoglycans in cancer,hsa05214:Glioma,hsa05215:Prostate cancer,hsa05218:Melanoma, |
| *IGF2* | insulin like growth factor 2(IGF2) | *Homo sapiens* | hsa05205:Proteoglycans in cancer, |
| *ITGB3* | integrin subunit beta 3(ITGB3) | *Homo sapiens* | hsa04015:Rap1 signaling pathway,hsa04145:Phagosome,hsa04151:PI3K-Akt signaling pathway,hsa04380:Osteoclast differentiation,hsa04510:Focal adhesion,hsa04512:ECM-receptor interaction,hsa04611:Platelet activation,hsa04640:Hematopoietic cell lineage,hsa04810:Regulation of actin cytoskeleton,hsa04919:Thyroid hormone signaling pathway,hsa05205:Proteoglycans in cancer,hsa05206:MicroRNAs in cancer,hsa05410:Hypertrophic cardiomyopathy (HCM),hsa05412:Arrhythmogenic right ventricular cardiomyopathy (ARVC),hsa05414:Dilated cardiomyopathy, |
| *KDR* | kinase insert domain receptor(KDR) | *Homo sapiens* | hsa04014:Ras signaling pathway,hsa04015:Rap1 signaling pathway,hsa04151:PI3K-Akt signaling pathway,hsa04370:VEGF signaling pathway,hsa04510:Focal adhesion,hsa05205:Proteoglycans in cancer, |
| *LDHA* | lactate dehydrogenase A(LDHA) | *Homo sapiens* | hsa00010:Glycolysis / Gluconeogenesis,hsa00270:Cysteine and methionine metabolism,hsa00620:Pyruvate metabolism,hsa00640:Propanoate metabolism,hsa01100:Metabolic pathways,hsa01130:Biosynthesis of antibiotics,hsa04922:Glucagon signaling pathway, |
| *MMP14* | matrix metallopeptidase 14(MMP14) | *Homo sapiens* | hsa04668:TNF signaling pathway,hsa04912:GnRH signaling pathway, |
| *MMP2* | matrix metallopeptidase 2(MMP2) | *Homo sapiens* | hsa04670:Leukocyte transendothelial migration,hsa04912:GnRH signaling pathway,hsa04915:Estrogen signaling pathway,hsa05200:Pathways in cancer,hsa05205:Proteoglycans in cancer,hsa05219:Bladder cancer, |
| *MMP7* | matrix metallopeptidase 7(MMP7) | *Homo sapiens* | hsa04310:Wnt signaling pathway, |
| *MMP9* | matrix metallopeptidase 9(MMP9) | *Homo sapiens* | hsa04668:TNF signaling pathway,hsa04670:Leukocyte transendothelial migration,hsa04915:Estrogen signaling pathway,hsa05161:Hepatitis B,hsa05200:Pathways in cancer,hsa05202:Transcriptional misregulation in cancer,hsa05205:Proteoglycans in cancer,hsa05206:MicroRNAs in cancer,hsa05219:Bladder cancer, |
| *MTHFR* | methylenetetrahydrofolate reductase(MTHFR) | *Homo sapiens* | hsa00670:One carbon pool by folate,hsa01100:Metabolic pathways,hsa01200:Carbon metabolism, |
| *MAPT* | microtubule associated protein tau(MAPT) | *Homo sapiens* | hsa04010:MAPK signaling pathway,hsa05010:Alzheimer's disease, |
| *MUC2* | mucin 2, oligomeric mucus/gel-forming(MUC2) | *Homo sapiens* | hsa05146:Amoebiasis, |
| *MSI1* | musashi RNA binding protein 1(MSI1) | *Homo sapiens* | hsa03015:mRNA surveillance pathway, |
| *MLH1* | mutL homolog 1(MLH1) | *Homo sapiens* | hsa03430:Mismatch repair,hsa03460:Fanconi anemia pathway,hsa05200:Pathways in cancer,hsa05210:Colorectal cancer,hsa05213:Endometrial cancer, |
| *PTEN* | phosphatase and tensin homolog(PTEN) | *Homo sapiens* | hsa00562:Inositol phosphate metabolism,hsa04068:FoxO signaling pathway,hsa04070:Phosphatidylinositol signaling system,hsa04071:Sphingolipid signaling pathway,hsa04115:p53 signaling pathway,hsa04150:mTOR signaling pathway,hsa04151:PI3K-Akt signaling pathway,hsa04510:Focal adhesion,hsa04931:Insulin resistance,hsa05161:Hepatitis B,hsa05200:Pathways in cancer,hsa05206:MicroRNAs in cancer,hsa05213:Endometrial cancer,hsa05214:Glioma,hsa05215:Prostate cancer,hsa05218:Melanoma,hsa05222:Small cell lung cancer,hsa05230:Central carbon metabolism in cancer, |
| *PIK3CA* | phosphatidylinositol-4,5-bisphosphate 3-kinase catalytic subunit alpha(PIK3CA) | *Homo sapiens* | hsa00562:Inositol phosphate metabolism,hsa04012:ErbB signaling pathway,hsa04014:Ras signaling pathway,hsa04015:Rap1 signaling pathway,hsa04024:cAMP signaling pathway,hsa04062:Chemokine signaling pathway,hsa04066:HIF-1 signaling pathway,hsa04068:FoxO signaling pathway,hsa04070:Phosphatidylinositol signaling system,hsa04071:Sphingolipid signaling pathway,hsa04150:mTOR signaling pathway,hsa04151:PI3K-Akt signaling pathway,hsa04152:AMPK signaling pathway,hsa04210:Apoptosis,hsa04370:VEGF signaling pathway,hsa04380:Osteoclast differentiation,hsa04510:Focal adhesion,hsa04550:Signaling pathways regulating pluripotency of stem cells,hsa04611:Platelet activation,hsa04620:Toll-like receptor signaling pathway,hsa04630:Jak-STAT signaling pathway,hsa04650:Natural killer cell mediated cytotoxicity,hsa04660:T cell receptor signaling pathway,hsa04662:B cell receptor signaling pathway,hsa04664:Fc epsilon RI signaling pathway,hsa04666:Fc gamma R-mediated phagocytosis,hsa04668:TNF signaling pathway,hsa04670:Leukocyte transendothelial migration,hsa04722:Neurotrophin signaling pathway,hsa04725:Cholinergic synapse,hsa04750:Inflammatory mediator regulation of TRP channels,hsa04810:Regulation of actin cytoskeleton,hsa04910:Insulin signaling pathway,hsa04914:Progesterone-mediated oocyte maturation,hsa04915:Estrogen signaling pathway,hsa04917:Prolactin signaling pathway,hsa04919:Thyroid hormone signaling pathway,hsa04923:Regulation of lipolysis in adipocytes,hsa04930:Type II diabetes mellitus,hsa04931:Insulin resistance,hsa04932:Non-alcoholic fatty liver disease (NAFLD),hsa04960:Aldosterone-regulated sodium reabsorption,hsa04973:Carbohydrate digestion and absorption,hsa05100:Bacterial invasion of epithelial cells,hsa05142:Chagas disease (American trypanosomiasis),hsa05146:Amoebiasis,hsa05160:Hepatitis C,hsa05161:Hepatitis B,hsa05162:Measles,hsa05164:Influenza A,hsa05166:HTLV-I infection,hsa05169:Epstein-Barr virus infection,hsa05200:Pathways in cancer,hsa05203:Viral carcinogenesis,hsa05205:Proteoglycans in cancer,hsa05210:Colorectal cancer,hsa05211:Renal cell carcinoma,hsa05212:Pancreatic cancer,hsa05213:Endometrial cancer,hsa05214:Glioma,hsa05215:Prostate cancer,hsa05218:Melanoma,hsa05220:Chronic myeloid leukemia,hsa05221:Acute myeloid leukemia,hsa05222:Small cell lung cancer,hsa05223:Non-small cell lung cancer,hsa05230:Central carbon metabolism in cancer,hsa05231:Choline metabolism in cancer, |
| *PLA2G2A* | phospholipase A2 group IIA(PLA2G2A) | *Homo sapiens* | hsa00564:Glycerophospholipid metabolism,hsa00565:Ether lipid metabolism,hsa00590:Arachidonic acid metabolism,hsa00591:Linoleic acid metabolism,hsa00592:alpha-Linolenic acid metabolism,hsa01100:Metabolic pathways,hsa04014:Ras signaling pathway,hsa04270:Vascular smooth muscle contraction,hsa04972:Pancreatic secretion,hsa04975:Fat digestion and absorption, |
| *PLAU* | plasminogen activator, urokinase(PLAU) | *Homo sapiens* | hsa04064:NF-kappa B signaling pathway,hsa04610:Complement and coagulation cascades,hsa05202:Transcriptional misregulation in cancer,hsa05205:Proteoglycans in cancer,hsa05206:MicroRNAs in cancer, |
| *PECAM1* | platelet and endothelial cell adhesion molecule 1(PECAM1) | *Homo sapiens* | hsa04514:Cell adhesion molecules (CAMs),hsa04670:Leukocyte transendothelial migration,hsa05144:Malaria, |
| *PDGFRB* | platelet derived growth factor receptor beta(PDGFRB) | *Homo sapiens* | hsa04010:MAPK signaling pathway,hsa04014:Ras signaling pathway,hsa04015:Rap1 signaling pathway,hsa04020:Calcium signaling pathway,hsa04151:PI3K-Akt signaling pathway,hsa04510:Focal adhesion,hsa04540:Gap junction,hsa04810:Regulation of actin cytoskeleton,hsa05166:HTLV-I infection,hsa05200:Pathways in cancer,hsa05206:MicroRNAs in cancer,hsa05214:Glioma,hsa05215:Prostate cancer,hsa05218:Melanoma,hsa05230:Central carbon metabolism in cancer,hsa05231:Choline metabolism in cancer, |
| *PROM1* | prominin 1(PROM1) | *Homo sapiens* | hsa05202:Transcriptional misregulation in cancer, |
| *PTGS2* | prostaglandin-endoperoxide synthase 2(PTGS2) | *Homo sapiens* | hsa00590:Arachidonic acid metabolism,hsa01100:Metabolic pathways,hsa04064:NF-kappa B signaling pathway,hsa04370:VEGF signaling pathway,hsa04668:TNF signaling pathway,hsa04723:Retrograde endocannabinoid signaling,hsa04726:Serotonergic synapse,hsa04913:Ovarian steroidogenesis,hsa04921:Oxytocin signaling pathway,hsa04923:Regulation of lipolysis in adipocytes,hsa05140:Leishmaniasis,hsa05200:Pathways in cancer,hsa05204:Chemical carcinogenesis,hsa05206:MicroRNAs in cancer,hsa05222:Small cell lung cancer, |
| *RRM1* | ribonucleotide reductase catalytic subunit M1(RRM1) | *Homo sapiens* | hsa00230:Purine metabolism,hsa00240:Pyrimidine metabolism,hsa00480:Glutathione metabolism,hsa01100:Metabolic pathways, |
| *RRM2* | ribonucleotide reductase regulatory subunit M2(RRM2) | *Homo sapiens* | hsa00230:Purine metabolism,hsa00240:Pyrimidine metabolism,hsa00480:Glutathione metabolism,hsa01100:Metabolic pathways,hsa04115:p53 signaling pathway, |
| *SEMA3B* | semaphorin 3B(SEMA3B) | *Homo sapiens* | hsa04360:Axon guidance, |
| *SIRT1* | sirtuin 1(SIRT1) | *Homo sapiens* | hsa04068:FoxO signaling pathway,hsa04152:AMPK signaling pathway,hsa04922:Glucagon signaling pathway,hsa05031:Amphetamine addiction,hsa05206:MicroRNAs in cancer, |
| *THBS1* | thrombospondin 1(THBS1) | *Homo sapiens* | hsa04015:Rap1 signaling pathway,hsa04115:p53 signaling pathway,hsa04145:Phagosome,hsa04151:PI3K-Akt signaling pathway,hsa04350:TGF-beta signaling pathway,hsa04510:Focal adhesion,hsa04512:ECM-receptor interaction,hsa05144:Malaria,hsa05205:Proteoglycans in cancer,hsa05206:MicroRNAs in cancer,hsa05219:Bladder cancer, |
| *TYMP* | thymidine phosphorylase(TYMP) | *Homo sapiens* | hsa00240:Pyrimidine metabolism,hsa00983:Drug metabolism - other enzymes,hsa01100:Metabolic pathways,hsa05219:Bladder cancer, |
| *TGFA* | transforming growth factor alpha(TGFA) | *Homo sapiens* | hsa04012:ErbB signaling pathway,hsa05200:Pathways in cancer,hsa05211:Renal cell carcinoma,hsa05212:Pancreatic cancer,hsa05214:Glioma,hsa05215:Prostate cancer,hsa05223:Non-small cell lung cancer, |
| *UMPS* | uridine monophosphate synthetase(UMPS) | *Homo sapiens* | hsa00240:Pyrimidine metabolism,hsa00983:Drug metabolism - other enzymes,hsa01100:Metabolic pathways, |
| *UPP1* | uridine phosphorylase 1(UPP1) | *Homo sapiens* | hsa00240:Pyrimidine metabolism,hsa00983:Drug metabolism - other enzymes,hsa01100:Metabolic pathways, |
| *VCAM1* | vascular cell adhesion molecule 1(VCAM1) | *Homo sapiens* | hsa04064:NF-kappa B signaling pathway,hsa04514:Cell adhesion molecules (CAMs),hsa04668:TNF signaling pathway,hsa04670:Leukocyte transendothelial migration,hsa05143:African trypanosomiasis,hsa05144:Malaria,hsa05166:HTLV-I infection, |
| *VEGFA* | vascular endothelial growth factor A(VEGFA) | *Homo sapiens* | hsa04014:Ras signaling pathway,hsa04015:Rap1 signaling pathway,hsa04066:HIF-1 signaling pathway,hsa04151:PI3K-Akt signaling pathway,hsa04370:VEGF signaling pathway,hsa04510:Focal adhesion,hsa05200:Pathways in cancer,hsa05205:Proteoglycans in cancer,hsa05206:MicroRNAs in cancer,hsa05211:Renal cell carcinoma,hsa05212:Pancreatic cancer,hsa05219:Bladder cancer,hsa05323:Rheumatoid arthritis, |
| *CDH17* | cadherin 17 (CDH17) | *Homo sapiens* | not annotated by DAVID6.7 |
| *CDX2* | caudal type homeobox 2 (CDX2) | *Homo sapiens* | not annotated by DAVID6.7 |
| *GADD45* | Growth Arrest and DNA Damage-inducible 45 (GADD45) | *Homo sapiens* | not annotated by DAVID6.7 |
| *LGALS4* | galectin 4 (LGALS4) | *Homo sapiens* | not annotated by DAVID6.7 |
| *LGR5* | leucine rich repeat containing G protein-coupled receptor 5 (LGR5) | *Homo sapiens* | not annotated by DAVID6.7 |
| *MGMT* | O-6-methylguanine-DNA methyltransferase (MGMT) | *Homo sapiens* | not annotated by DAVID6.7 |
| *MIA* | melanoma inhibitory activity (MIA) | *Homo sapiens* | not annotated by DAVID6.7 |
| *MMP10* | matrix metallopeptidase 10 (MMP10) | *Homo sapiens* | not annotated by DAVID6.7 |
| *MMP11* | matrix metallopeptidase 11 (MMP11) | *Homo sapiens* | not annotated by DAVID6.7 |
| *MUC13* | mucin 13, cell surface associated (MUC13) | *Homo sapiens* | not annotated by DAVID6.7 |
| *OLFM4* | olfactomedin 4 (OLFM4) | *Homo sapiens* | not annotated by DAVID6.7 |
| *PDL1* | Programmed death-ligand 1 (PDL1) | *Homo sapiens* | not annotated by DAVID6.7 |
| *PDL2* | Programmed death-ligand 2 (PDL2) | *Homo sapiens* | not annotated by DAVID6.7 |
| *REG4* | regenerating family member 4 (REG4) | *Homo sapiens* | not annotated by DAVID6.7 |
| *RUNX3* | runt related transcription factor 3 (RUNX3) | *Homo sapiens* | not annotated by DAVID6.7 |
| *SPARC* | secreted protein acidic and cysteine rich (SPARC) | *Homo sapiens* | not annotated by DAVID6.7 |
| *TM9SF3* | transmembrane 9 superfamily member 3 (TM9SF3) | *Homo sapiens* | not annotated by DAVID6.7 |
| *TOP1* | topoisomerase (DNA) I (TOP1) | *Homo sapiens* | not annotated by DAVID6.7 |
| *TOP2A* | topoisomerase (DNA) II alpha (TOP2A) | *Homo sapiens* | not annotated by DAVID6.7 |
| *TSPAN8* | tetraspanin 8 (TSPAN8) | *Homo sapiens* | not annotated by DAVID6.7 |
| *VSNL1* | visinin like 1 (VSNL1) | *Homo sapiens* | not annotated by DAVID6.7 |
| *ZDHHC14* | zinc finger DHHC-type containing 14 (ZDHHC14) | *Homo sapiens* | not annotated by DAVID6.7 |
